# Polydatin and Nicotinamide Rescue the Cellular Phenotype of Mitochondrial Diseases by Mitochondrial Unfolded Protein Response (mtUPR) Activation

**DOI:** 10.3390/biom14050598

**Published:** 2024-05-18

**Authors:** Paula Cilleros-Holgado, David Gómez-Fernández, Rocío Piñero-Pérez, José Manuel Romero Domínguez, Marta Talaverón-Rey, Diana Reche-López, Juan Miguel Suárez-Rivero, Mónica Álvarez-Córdoba, Ana Romero-González, Alejandra López-Cabrera, Marta Castro De Oliveira, Andrés Rodríguez-Sacristan, José Antonio Sánchez-Alcázar

**Affiliations:** 1 Centro Andaluz de Biología del Desarrollo (CABD-CSIC-Universidad Pablo de Olavide), 41013 Sevilla, Spain; pcilhol@upo.es (P.C.-H.); dgomfer1@acu.upo.es (D.G.-F.); rpieper@alu.upo.es (R.P.-P.); jmromdom@upo.es (J.M.R.D.); mtalrey@upo.es (M.T.-R.); dreclop@alu.upo.es (D.R.-L.); juanmiguel.suarez@inibica.es (J.M.S.-R.); malvcor@upo.es (M.Á.-C.); aromgon1@upo.es (A.R.-G.); alopcab2@alu.upo.es (A.L.-C.); 2 Neuropediatria, Neurolinkia, C. Jardín de la Isla, 8, Local 4 y 5, 41014 Sevilla, Spain; martadecastro@neurolinkia.com; 3 FEA Pediatría, Centro Universitario Hospitalar de Faro, R. Leão Penedo, 8000-386 Faro, Portugal; 4 Neuropediatría, Servicio de Pediatría, Hospital Universitario Virgen Macarena, 41009 Sevilla, Spain; arodriguezsacristan@us.es; 5 Departamento de Farmacología, Radiología y Pediatría, Facultad de Medicina, Universidad de Sevilla, 41009 Sevilla, Spain

**Keywords:** mitochondrial diseases, *GFM1*, EF-G1, fibroblasts, direct reprogramming, induced neurons, mitochondria, mtUPR, treatment

## Abstract

Primary mitochondrial diseases result from mutations in nuclear DNA (nDNA) or mitochondrial DNA (mtDNA) genes, encoding proteins crucial for mitochondrial structure or function. Given that few disease-specific therapies are available for mitochondrial diseases, novel treatments to reverse mitochondrial dysfunction are necessary. In this work, we explored new therapeutic options in mitochondrial diseases using fibroblasts and induced neurons derived from patients with mutations in the *GFM1* gene. This gene encodes the essential mitochondrial translation elongation factor G1 involved in mitochondrial protein synthesis. Due to the severe mitochondrial defect, mutant *GFM1* fibroblasts cannot survive in galactose medium, making them an ideal screening model to test the effectiveness of pharmacological compounds. We found that the combination of polydatin and nicotinamide enabled the survival of mutant *GFM1* fibroblasts in stress medium. We also demonstrated that polydatin and nicotinamide upregulated the mitochondrial Unfolded Protein Response (mtUPR), especially the SIRT3 pathway. Activation of mtUPR partially restored mitochondrial protein synthesis and expression, as well as improved cellular bioenergetics. Furthermore, we confirmed the positive effect of the treatment in *GFM1* mutant induced neurons obtained by direct reprogramming from patient fibroblasts. Overall, we provide compelling evidence that mtUPR activation is a promising therapeutic strategy for *GFM1* mutations.

## 1. Introduction

Mitochondrial diseases encompass a heterogeneous group of genetic, chronic, and progressive pathologies characterized by mitochondrial dysfunction and the overproduction of reactive oxygen species (ROS) due to defects in oxidative phosphorylation (OXPHOS) or proteins involved in mitochondrial function [1]. These diseases, considered rare with a prevalence estimated at 1 in 5000, result from mutations in nuclear DNA (nDNA) or mitochondrial DNA (mtDNA) [2]. From a clinical perspective, mitochondrial diseases impact various organs and systems, with a notable influence on the nervous and muscular systems due to their high energy requirements. Consequently, common symptoms include exercise intolerance, muscle weakness, neurosensory hearing loss, neurodegeneration, axonal neuropathy, diabetes mellitus, and gastrointestinal disorders [3].

While many mitochondrial illnesses stem from mutations in genes encoding mitochondrial electron transport chain (mtETC) proteins, there are instances of mutations in genes encoding proteins responsible for other mitochondrial processes, including maintenance, transcription, or translation [4]. Mitochondria have their own translation system, which is different from its cytoplasmatic counterpart, yet follows the same initiation, elongation, termination, and ribosome-recycling steps [5]. In the elongation step, three factors play a role, including the mitochondrial translation elongation factor G1 (EF-G1), encoded by the nuclear *G elongation Factor Mitochondrial 1* (*GFM1*) gene. EF-G1 is a GTPase that catalyzes the translocation step in which it binds to the aminoacyl site (A) and induces ribosomal translocation by promoting A-site-bound peptidyl-tRNA and peptidyl site (P)-bound deacylated-tRNA to move to the P and exit site (E), respectively [6]. Mutations in the *GFM1* gene typically result in a disease called combined oxidative phosphorylation deficiency type 1, an autosomal recessive pathology. This condition is characterized by disruptions in mitochondrial-encoded protein synthesis, leading to early onset severe encephalopathy, dystonia, feeding difficulties, liver failure, increased lactate levels, and other symptoms [7].

Regrettably, effective treatments for most mitochondrial diseases are currently unavailable, underscoring the pressing need to explore novel therapeutic approaches that can enhance the quality of life for patients.

In a recent study, our group demonstrated significant progress by activating the mitochondrial Unfolded Protein Response (mtUPR) through the administration of tetracyclines, a class of broad-spectrum antibiotics. This approach notably improved the pathophysiology of cellular models with *GFM1* mutations [8]. In another publication, we expanded on these findings, showcasing that mtUPR activation, achieved using pterostilbene in conjunction with mitochondrial cofactors, offered substantial improvements in mitochondrial pathophysiology. This positive outcome was observed in both fibroblasts and induced neurons derived from patients with mitochondrial diseases caused by mutations in nDNA or mtDNA [9].

mtUPR was initially described in mammals [10] as a compensatory mechanism for mitochondria [11,12]. Originally defined as a transcriptional process, mtUPR upregulated the expression of mitochondrial proteases and chaperones in response to elevated levels of unfolded or misfolded proteins within mitochondria [13]. Over time, this stress response has recently emerged as a potential therapeutic target for a wide selection of diseases, including mitochondrial diseases [8,9,14,15].

Several compounds have been identified as mtUPR activators [16]. Among them is nicotinamide [17,18], which enhances nicotinamide adenine dinucleotide (NAD^+^) biosynthesis. NAD^+^ serves as a key cofactor that modulates various aspects of mitochondrial metabolism, mitonuclear protein imbalance, or sirtuin family activity [19]. Moreover, an association between mtUPR and mitochondrial biogenesis has been observed in conditions of increased NAD^+^ levels, as this cofactor activates sirtuins. Sirtuins are responsible for the activation of Forkhead box O3 (FOXO3a), a factor involved in mtUPR, and peroxisome proliferator-activated receptor γ co-activator 1α (PGC1α), which participates in mitochondrial biogenesis [15]. The activation of these factors further contributes to the recovery of mitochondrial function.

However, polydatin, a glucoside derivative of resveratrol, has not been explicitly described as a direct activator of mtUPR *per se*. Instead, it has primarily been used for its well-established antioxidant [20], anti-inflammatory [21], and anti-apoptotic [22] properties. Nevertheless, some authors have noted its effect as an activator of SIRT3 [23], prompting us to include it among the compounds explored for mtUPR activation.

In this work, we employed a cocktail based on polydatin and nicotinamide as mtUPR activators. This approach was undertaken to improve pathophysiological features in fibroblasts and induced neurons derived from three patients with mutations in the *GFM1* gene.

## 2. Materials and Methods

### 2.1. Reagents

Anti-EF-G1 (ab173529), anti-mitochondrially encoded NADH:Ubiquinone Oxidoreductase Core Subunit 3 (Mt-ND3) (ab170681), anti-mitochondrially encoded Cytochrome C Oxidase Subunit II (MtCO2) (ab79393), anti-Cytochrome C Oxidase Subunit IV (COX IV) (ab14744), anti-ATP synthase F1 subunit alpha (ATP5F1A) (ab14748), anti-voltage-dependent anion channel (VDAC) (ab14734), anti-sirtuin 1 (SIRT1) (ab110304), anti-Lon peptidase 1 (Lonp1) (ab103809), anti-nuclear respiratory factor 2 (Nrf2) (ab62352), anti-Activating Transcription Factor 5 (ATF5) (ab184923), anti-PGC1α (ab191838), anti-manganese superoxide dismutase (MnSOD) (ab68155), Goat Anti-Rabbit IgG H&L (HRP) (ab6721), Rabbit Anti-Mouse IgG H&L (HRP) (ab6728), and Rabbit Anti-Goat IgG H&L (HRP) (ab6741) were purchased from Abcam (Cambridge, UK).

Anti-NADH:Ubiquinone Oxidoreductase Core Subunit S1 (NDUFS1) (PA5-22309), anti-sirtuin 3 (SIRT3) (PA5-13222), anti-heat shock protein 60 (Hsp60) (MA3-012), anti-heat shock protein 70 (Hsp70) (MA3-028), Mitotracker^TM^ Red CMXRos (M46752), Mitotracker^TM^ Deep Red FM (M22426), 4′,6-diamidino-2-phenylindole (DAPI) (D1306) and Bovin Serum Albumine (BSA) (BP9702-100) were purchased from Invitrogen^TM^/Molecular probes (Eugene, OR, USA).

Anti-succinate dehydrogenase complex iron-sulfur subunit B (SDHB) (sc-271548), anti-FOXO3a (sc-48348), anti-Tau (sc-32240), D-galactose (sc-202564), rotenone (sc-203242), oligomycin (sc-203342), Carbonyl cyanide 4-(trifluoromethoxy)phenylhydrazone (FCCP) (sc-203578), L-glutamine (sc-391013), chloramphenicol (sc-3594), 4-(2-Hydroxyethyl)-1-piperazine ethanesulfonic acid (HEPES) (sc-29097), and antimycin A (sc-202467A) were purchased from Santa Cruz Biotechnology (Dallas, TX, USA).

Anti-Activating Transcription Factor 4 (ATF4) (11815S), anti-eukaryotic translation initiation factor 2A (eif2α) (5324S), anti-phosphorylated-eif2α (P-eif2α) (9721S), and anti-mitochondrial transcription factor A (TFAM) (7495S) were purchased from Cell Signaling (Danvers, MA, USA).

Paraformaldehyde (PFA) (158127), L-cysteine (168149), nicotinamide (N7004), D-glucose (G7879), cycloheximide (01810), sodium pyruvate (P5280), and dimethyl sulfoxide (DMSO) (17093) were purchased from Sigma-Aldrich (Saint Louis, MO, USA). Antibody anti-actin (MBS448085) was purchased from MyBioSource (San Diego, CA, USA). Anti-nuclear respiratory factor 1 (Nrf1) (NBP1-778220) was purchased from Novus Biologicals (Móstoles, Madrid, Spain). Anti-phosphorylated-PGC1α (P-PGC1α) (AF6650) was purchased from RD Systems (Minneapolis, MN, USA). Polydatin (21246) was purchased from Cayman-Chemicals (Ann Arbor, MI, USA). 3-(1H-1,2,3-triazol-4-yl)pyridine (3-TYP) (HY-108331) was purchased from MedChemExpress (Sollentuna, Sweden). Phosphate-buffered saline (PBS) (102309) was purchased from iNtRON Biotechnology (Seongnam, Republic of Korea).

### 2.2. Ethical Statements

The present study received approval from the ethical committees of the Hospital Universitario Virgen Macarena and Hospital Universitario Virgen del Rocío (Seville, Spain), Mitocure Code 0543-N-16, dated 11-08-2016, according to the International Conference on Harmonisation and Good Clinical Practice Guidelines as well as the Declaration of Helsinki principles.

### 2.3. Fibroblast Cultures

We cultured fibroblasts derived from skin biopsies of three mitochondrial patients (P1, P2, and P3) harboring the following mutations:

- P1, P2 (brothers): heterozygous mutation c.179C>G, p. (Thr60Ser) in exon 2 (NM_001308164.1, OMIM 606639), and c.2068C>T, p. (Arg690Cys) in exon 17 (NM_001308164.1, OMIM 606639) of the *GFM1* gene.
- P3: heterozygous mutation c.1404delA, p. (Gly469Valfs*84) in exon 12 (NM_024996.5, OMIM 606639), and c.2011C>T, p. (Arg671Cys) in exon 16 (NM_024996.5, OMIM 606639) of the *GFM1* gene.

We used control lines of primary human skin fibroblasts derived from healthy volunteer donors (C1, C2 and C3). These control cells were sex- and age-matched.

Patient and control cells were obtained following the Helsinki Declarations of 1964 (revised in 2001). Fibroblasts were cultured in Dulbecco’s modified Eagle’s medium (DMEM) (Gibco^TM^, Waltham, MA, USA) with 10% Fetal Bovine Serum (FBS) (Gibco^TM^, Waltham, MA, USA) and 1% penicillin/streptomycin (Sigma-Aldrich, Saint Louis, MO, USA) at 37 °C and 5% CO_2_. Cells were treated with polydatin and nicotinamide at 10 µM for seven days. All experiments were performed with cell cultures with a passage number lower than 10.

### 2.4. Drug Screening

Drug screening was assessed by culturing the cells in a restrictive medium with galactose as the unique carbon source. The galactose medium was prepared using DMEM no glucose (Invitrogen^TM^ Molecular Probes, Eugene, OR, USA) supplemented with 20 mM D-galactose, 15 mM HEPES, 1% penicillin/streptomycin, and 10% FBS. Cells were seeded in 24-well plates in DMEM 1 g/L glucose and treated with different compounds. After 3 days, the glucose medium was removed and changed to galactose medium, with treatments reapplied. Images and cell counting were obtained immediately (T0) and 72 h after the shift to galactose medium (T72) using the BioTek^TM^ Cytation 1 Cell Imaging Multi-Mode Reader (Biotek, Winooski, VT, USA). The proliferation ratio was obtained by dividing the number of cells at T72 by the number of cells at T0. Proliferation ratio values above 1 were considered as cell proliferation, while values below 1 were considered as cell death, and a value of 1 indicated cell survival. Positive treatments were those that allowed the survival of patient cells in the glucose-free galactose medium, with the cocktail of polydatin and nicotinamide at 10 µM selected, as the others failed to make mutant cells survive and were deemed negative. Cell viability was confirmed by trypan blue dye exclusion.

This screening was repeated using 3-TYP, a specific inhibitor of SIRT3. The concentration employed was 32 nM, as this compound exhibits an IC_50_ (half-maximal inhibitory concentration) of 16 nM for SIRT3, requiring a higher concentration for SIRT1 (IC_50_ = 88 nM) and SIRT2 (IC_50_ = 92 nM). This ensures the specific inhibition of SIRT3 without affecting other sirtuins. Cells were initially seeded in glucose medium and treated with polydatin and nicotinamide at 10 µM, along with 3-TYP at 32 nM for a duration of 3 days. Subsequently, the glucose medium was replaced with galactose medium, treatments were renewed, and images were captured using the same methodology as in the previous screening.

### 2.5. Quantitative Real-Time PCR

The expression of the *GFM1* gene was assessed in both control and patient fibroblasts through quantitative real-time PCR, utilizing mRNA extracts. Total RNA extraction was carried out using the RNeasy Mini Kit (74104, Qiagen, Venlo, The Netherlands), followed by cDNA synthesis from 1 µg of RNA using the iScript cDNA KIT (170-8891, BioRad, Hercules, CA, USA). After that, qPCR was conducted employing standard procedures and the SYBR Green Protocol. *GFM1* primers were 5′-CCGGAGACATCTGTGCATTG-3′ (Forward primer) and 5′-CATAGAAAGGCCGCTGTTGG-3′ (Reverse primer). Actin was used as a housekeeping control gene, and the primers were 5′-AGAGCTACGAGCTGCCTGAC-3′ (Forward primer) and 3′-AGCACTGTGTTGGCGTACAG-5′ (reverse primer). Primer design was facilitated using the online tool Primer3 (https://primer3.ut.ee/, accessed on 3 March 2021).

### 2.6. Immunoblotting

Western blot analysis was conducted following standard methods. After protein transfer, nitrocellulose membranes (1620115, BioRad, Hercules, CA, USA) were blocked with 5% BSA in TTBS (blocking solution) and then incubated with primary antibodies at an appropriate dilution range (1:500–1:2000) in the blocking solution overnight at 4 °C. Then, membranes were incubated with the corresponding secondary antibody coupled to horseradish peroxidase (HRP) at a dilution range of 1:2500–1:10,000 for 1 h at room temperature. Protein bands were visualized using the Chemidoc^TM^ MP Imaging System (BioRad, Hercules, CA, USA) with the Immun Star HRP substrate kit (1705061, BioRad, Hercules, CA, USA). The obtained results were normalized to the mean expression levels of control cells and the housekeeping protein actin.

In cases where proteins were sufficiently separated, membranes were cut, and each piece was incubated with a different antibody. The revealed membranes were then analyzed by ImageLab^TM^ software version 5.2.1 (BioRad, Hercules, CA, USA).

### 2.7. Immunofluorescence Microscopy

For immunofluorescence microscopy, cells were seeded on 1 mm glass coverslips (631-1331, Menzel-Gläser, ThermoFisher Scientific, Waltham, MA, USA) for 72 h in DMEM glucose, with or without the supplementation of treatment. The cells were fixed with 4% PFA for 10 min and then permeabilized with either 0.01% Triton X-100 or saponin for an additional 10 min. After that, the cells were incubated with 5% donkey serum in PBS (blocking solution) for 1 h. Primary antibodies, appropriately diluted in the blocking solution (1:100–1:400), were then incubated overnight at 4 °C. Following primary antibody incubation, cells underwent two washes with PBS 1× and were incubated for 2 h at room temperature with the corresponding secondary antibodies, diluted 1:200 in blocking solution. After that, cells were washed twice with PBS 1× and incubated with 1 µg/mL of DAPI for 10 min. Finally, after 5 washes with PBS 1×, coverslips were mounted on microscope slides using 10 µL of Mowiol. Images were acquired using either a DeltaVision system (Applied Precision; Issaqua, WA, USA) with an Olympus IX-71 fluorescent microscope (Olympus Corporation, Tokyo, Japan) with a 40× objective or a Zeiss880 ‘Airyscan’ confocal microscope (Carl Zeiss AG, Oberkochen, Germany) with a 63× objective. Fiji-ImageJ software version 1.53.2 was used for image analysis. The microscope settings were consistently maintained across each experiment.

### 2.8. Measurement of Mitochondrial Membrane Potential

The measurement of mitochondrial membrane potential was conducted using Mitotracker^TM^ CMXRos, a fluorescent dye sensitive to mitochondrial membrane potential. Untreated and treated cells were seeded on 1 mm glass coverslips in DMEM glucose for three days. Subsequently, cells were stained with 100 nM Mitotracker^TM^ Red CMXRos for 45 min at 37 °C before fixation. Following staining, cells were washed twice with PBS 1x and fixed with PFA 4% for 10 min. Then, cells were incubated with 1 µg/mL of DAPI for 10 min. Finally, after 5 washes with PBS 1×, the coverslips were mounted on microscope slides using 10 µL of Mowiol. Images were captured using a DeltaVision system (Applied Precision; Issaqua, WA, USA) with an Olympus IX-71 fluorescent microscope (Olympus Corporation, Tokyo, Japan) with a 40× objective and analyzed using Fiji-ImageJ software version 1.53.2. The microscope settings were consistently maintained in each experiment, and mitochondrial membrane potential was calculated based on fluorescence intensity. To determine the ratio between rounded and tubular mitochondria, Fiji-ImageJ software version 1.53.2 was used, categorizing rounded mitochondria as those with a size of 0.2–0.5 µm and tubular mitochondria as those exceeding 0.5 µm in size.

### 2.9. Measurement of Protein Synthesis

The measurement of protein synthesis was assessed using the Click-iT^®^ HPG 488 Alexa Fluor Protein Synthesis Assay Kit (C10428, Life technologies, Carlsbad, CA, USA). Untreated and treated cells were seeded on 1 mm glass coverslips for 72 h in DMEM glucose. After that, cells were incubated with 100 nM Mitotracker^TM^ Deep Red FM for 45 min at 37 °C. To observe and measure cytosolic and mitochondrial protein synthesis, cells were treated with chloramphenicol 150 µg/mL for 30 min or cycloheximide 50 µg/mL for 20 min, respectively. Next, following the manufacturer’s protocol, cells were incubated with the alkyne-containing non-canonical amino acid L-homoproparglyglycine (HPG) for 30 min in a methionine-free medium supplemented with 200 µM L-cystine, 10 mM HEPES, and 2 mM L-glutamine. Finally, cells were incubated with 1 µg/mL of DAPI for 10 min, washed 5 times with PBS 1×, and the coverslips were mounted on microscope slides using 10 µL of Mowiol. Images were taken using a Zeiss880 ‘Airyscan’ confocal microscope (Carl Zeiss AG, Okerkochen, Germany) with a 63× objective with zoom of 2, and analysis was performed using ImageJ-Fiji software version 1.53.2. The microscope settings were consistently maintained in each experiment.

### 2.10. Bioenergetics

Key parameters of mitochondrial respiration were assessed using a Mitostress test assay conducted on the XF24 extracellular flux analyzer (Seahorse Bioscience, Billerica, MA, USA) according to the manufacturer’s instructions. Cells were seeded in XF24 cell culture plates at a density of 1.5 × 10^4^ cells/well with 250 µL of DMEM glucose medium and incubated at 37 °C and 5% CO_2_ for 24 h. After that, 200 µL of DMEM medium was removed, and cells were washed twice with 500 µL of assay XF base medium supplemented with 10 mM D-glucose, 1 mM L-glutamine, and 1 mM sodium pyruvate. Finally, 450 µL of assay medium were added to achieve a final volume of 500 µL.

Mitochondrial function was measured by sequential injections of four compounds affecting bioenergetics: 1 µM oligomycin (complex V inhibitor), 1 µM rotenone (complex I inhibitor), 2 µM FCCP (uncoupling agent), and 2.5 µM antimycin A (complex III inhibitor). This assay facilitated the measurement of key parameters, including basal respiration, maximal respiration, spare respiratory capacity, and ATP production. Normalization was carried out by cell counting using the BioTek^TM^ Cytation 1 Cell Imaging Multi-Mode Reader (Biotek, Winooski, VT, USA).

### 2.11. Measurement of Mitochondrial Complex Activity

The activity of mitochondrial complex I and IV was assessed using the Complex I (ab109720)/Complex IV (ab109876) Enzyme Activity Dipstick Assay Kit (Abcam, Cambridge, UK), according to the manufacturer’s instructions starting from cellular pellets. Signal intensity was acquired using the Chemidoc^TM^ MP Imaging System and analyzed using ImageLab^TM^ software version 5.2.1 (BioRad, Hercules, CA, USA).

### 2.12. Measurement of NAD^+^/NADH Levels

NAD^+^/NADH levels were quantified using the NAD^+^/NADH Colorimetric Assay Kit (ab65348, Abcam, Cambridge, UK) protocol, starting from cellular pellets. The color intensity was measured using a POLARstar Omega plate reader (BMG Labtech, Offenburg, Germany).

### 2.13. Measurement of SIRT3 Activity

The isolation of the mitochondrial fraction was conducted using the Mitochondrial Isolation Kit for Cultured Cells (ab110170, Abcam, Cambridge, UK). Subsequently, SIRT3 activity in mitochondrial fractions was assessed using the SIRT3 Fluorometric Activity Assay Kit (ab156067, Abcam, Cambridge, UK), following the manufacturer’s instructions. Fluorescence intensity was quantified using a POLARstar Omega plate reader (BMG Labtech, Offenburg, Germany).

### 2.14. Direct Reprogramming

We generated induced neurons (iNs) from both control and mutant fibroblasts through direct reprogramming [24,25,26]. Cells were initially seeded in μ-Slide 4 Ibidi plates in DMEM + Glutamax medium (10566016, ThermoFisher Scientific, Waltham, MA, USA), supplemented with 1% penicillin/streptomycin and 10% FBS for 24 h. Next, the cells were infected with one-single lentiviral vector containing two shRNAs against the REST complex and two neural lineage-specific transcription factors (*Achaete-Scute Family BHLH Transcription Factor 1* (*ASCL1*)*, POU class 3 homeobox 2* (*BRN2*)), obtained as previously described [27], at a multiplicity of infection of 30. Plasmids were provided as a gift from Dr. Malin Parmar (Developmental and Regenerative Neurobiology, Lund University, Sweden). The following day, DMEM + Glutamax medium was replaced with fresh DMEM, and 48 h later, neural differentiation medium (supplemented NDiff27 (Y40002, Takara-Clontech, San Jose, CA, USA) as described before [24]. Half of the neural differentiation medium was changed every 2–3 days. At 18 days post-cellular infection, the medium was replaced with NDiff27 only supplemented with growth factors. On day 21, cells were treated with polydatin and nicotinamide at 10 µM for seven days. By day 28 post-infection, neuronal purity and conversion efficiency were calculated, considering Tau+ cells as iNs.

### 2.15. Statistical Analysis

For datasets with a sample size exceeding 30 (n > 30), parametric statistical methods were employed, specifically one-way ANOVA, for comparing statistical differences among more than two groups. In cases where the sample size was below (n < 30), non-parametric methods such as the Mann–Whitney test were utilized for pairwise comparisons between two groups, while the Kruskal–Wallis test was employed for comparing multiple groups. All statistical analyses were performed using GraphPad Prism 9.4.1 (GraphPad Software, San Diego, CA, USA). A significance threshold of 0.05 or lower (*p* ≤ 0.05) was considered statistically significant.

## 3. Results

### 3.1. Polydatin and Nicotinamide Supplementation Enabled GFM1 Fibroblast Survival in Galactose Medium

The survival of *GFM1* mutant fibroblasts in galactose medium poses a unique challenge due to the severe mitochondrial dysfunction characteristic of these cells, rendering them suitable candidates for pharmacological screening under nutritional stress conditions [28]. In the presence of glucose, cells typically utilize glycolysis and OXPHOS for energy production. However, when cultured in galactose as the sole carbon source, cells are compelled to rely solely on OXPHOS. This shift in energy source leads to the inability of *GFM1* fibroblasts to survive in this nutritional stress medium.

**Figure 1:**
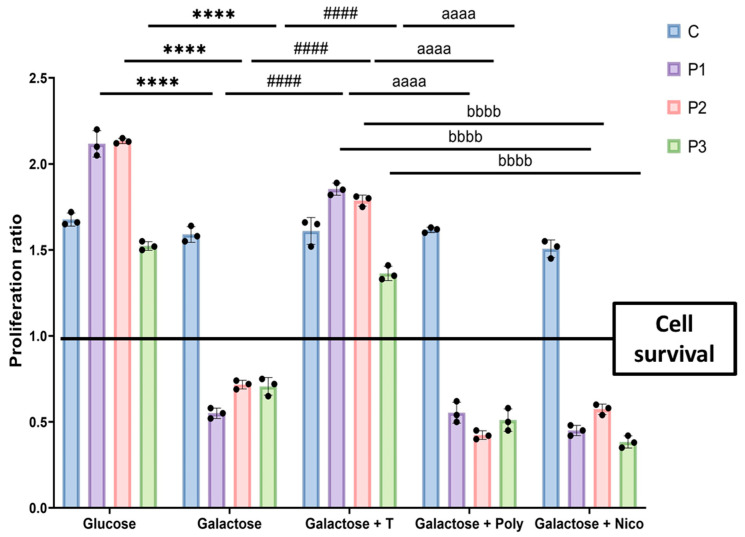
Proliferation ratio quantification of galactose drug screening. Control (C) and mutant (P1, P2, P3) cells were initially seeded in glucose medium and treated with polydatin and nicotinamide at 10 μM (T), polydatin at 10 µM (Poly), or nicotinamide at 10 µM (Nico). After 3 days, the glucose medium was changed to galactose medium, and treatments were refreshed. Cell counting was obtained immediately (T0) and 72 h later (T72) using the BioTek Cytation 1 Cell Imaging Multi-Mode Reader. The proliferation ratio was determined by dividing the number of cells at T72 by the number of cells at T0. Results equal to 1 indicate cell survival, values greater than 1 denote cell proliferation, and values below 1 signify cell death. C represents the mean of C1, C2, and C3 cells. Data represent the mean ± SEM of three independent experiments. **** *p* < 0.0001 between mutant cells in glucose and galactose medium. ^####^ *p* < 0.0001 between untreated and cocktail-treated mutant fibroblasts in galactose medium. ^aaaa^ *p* < 0.0001 between untreated and polydatin-treated mutant fibroblasts in galactose medium. ^bbbb^ *p* < 0.0001 between untreated and nicotinamide-treated mutant fibroblasts in galactose medium. Refer to additional file: Figure S1 for representative images of the drug screening assay.

Control and mutant fibroblasts were initially seeded in glucose medium for three days, with or without the supplementation of various compounds. Subsequently, the glucose medium was replaced with galactose medium, and treatments were refreshed. Control cells exhibited normal growth in both glucose and galactose media. In contrast, mutant cells displayed normal growth rates in glucose medium but succumbed to cell death in galactose medium after 72 h. Remarkably, when mutant fibroblasts were treated with a cocktail of 10 µM polydatin and 10 μM nicotinamide, they exhibited survival in the nutritional stress medium. As anticipated, control cells maintained their proliferation ratio with no discernible changes when cultured in galactose medium with the treatment. However, when we treated mutant fibroblasts with either polydatin or nicotinamide at 10 µM individually, cells experienced cell death in galactose medium, suggesting that the combination of both compounds is needed for the positive effect (Figure 1). Consequently, the supplementation of polydatin and nicotinamide was selected for subsequent assays.

### 3.2. Polydatin and Nicotinamide Treatment Increased Mutant GFM1 Transcript and Protein Expression Levels and Improved Mitochondrial Protein Content

**Figure 2:**
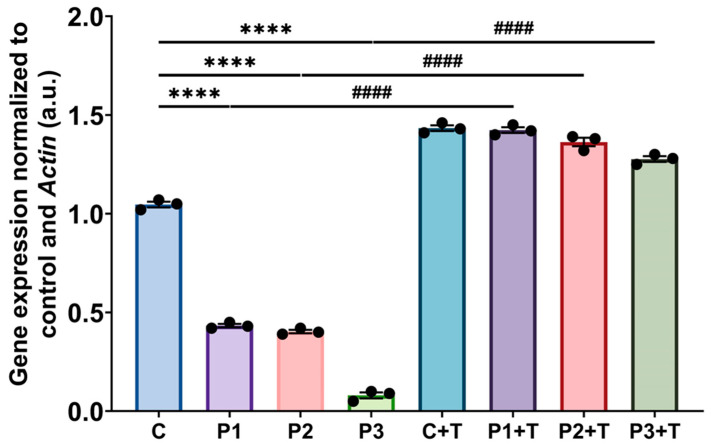
*GFM1* transcript expression levels in untreated and treated mutant (P1, P2, P3) and control (C) fibroblasts. Cells were treated with polydatin and nicotinamide at 10 µM for seven days (+T). *Actin* served as the housekeeping gene. C represents the mean of C1, C2, and C3 cells. Data represent the mean ± SEM of three independent experiments. **** *p* < 0.0001 between control and patient fibroblasts. ^####^ *p* < 0.0001 between untreated and treated mutant cells.

Next, we investigated the impact of polydatin and nicotinamide treatment on pathophysiological alterations in mutant fibroblasts (P1, P2, and P3). The initial examination of *GFM1* transcript levels by qPCR revealed a significant decrease in patient fibroblasts, a trend that was effectively reversed following supplementation with polydatin and nicotinamide (Figure 2).

**Figure 3:**
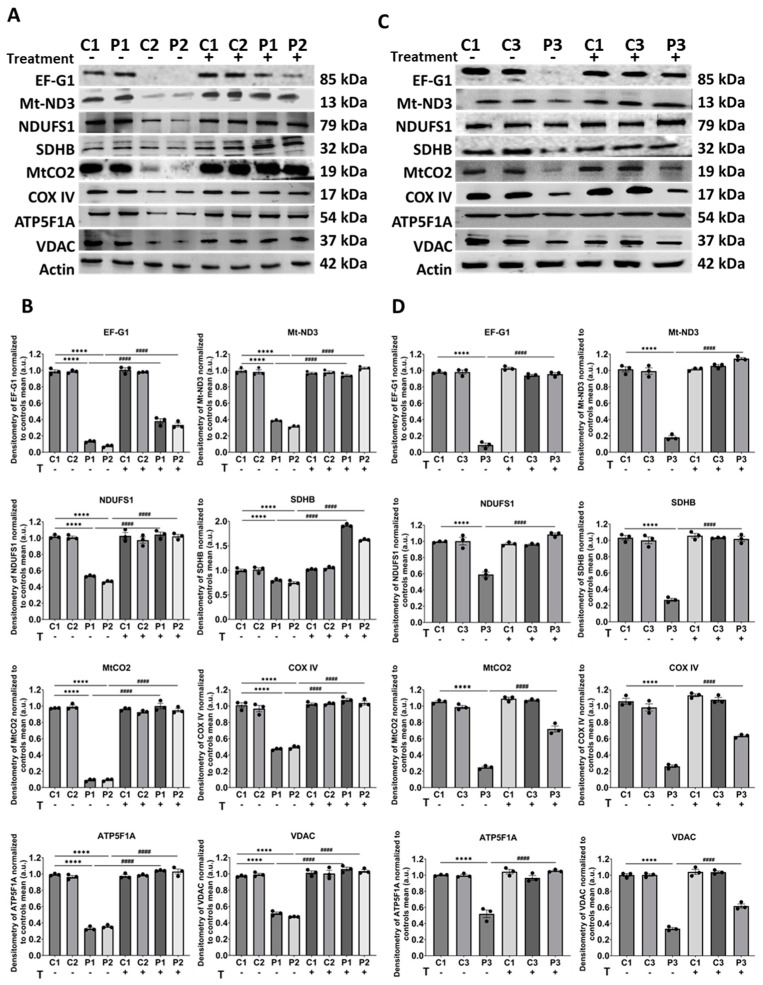
Expression levels of mitochondrial proteins in control and mutant cells with and without treatment. Cells were either untreated or treated with polydatin and nicotinamide at 10 µM for seven days. (**A**) Immunoblotting analysis of the mutant protein (EF-G1), along with other mitochondrial proteins such as Mt-ND3 and NDUFS1 (complex I), SDHB (complex II), MtCO2 and COX IV (complex IV), and ATP5F1A (complex V) in control (C1, C2) and mutant (P1, P2) cells. VDAC was used as a mitochondrial mass marker, and actin served as the loading control. (**B**) Band densitometry of Western Blot data, referred to actin levels and normalized to the mean of controls. (**C**) Immunoblotting analysis of the mutant protein (EF-G1), along with other mitochondrial proteins such as Mt-ND3 and NDUFS1 (complex I), SDHB (complex II), MtCO2 and COX IV (complex IV), and ATP5F1A (complex V) in control (C1, C3) and patient (P3) cells. Original images can be found in Supplementary Materials. (**D**) Band densitometry of Western blot data, referred to actin levels and normalized to the mean of controls. Data represent the mean ± SEM of three independent experiments. **** *p* < 0.0001 between control and patient fibroblasts. ^####^ *p* < 0.0001 between untreated and treated mutant fibroblasts. a.u.: arbitrary units.

In addition, considering the compromised mitochondrial protein synthesis associated with *GFM1* mutations, we extended our analysis to include Western blot examination of the mutant protein (EF-G1) and various mitochondrial respiratory complex proteins, including those from complex I (Mt-ND3 and NDUFS1), complex II (SDHB), complex IV (MtCO2 and COX IV), and complex V (ATP5F1A). The protein expression levels of VDAC, serving as a mitochondrial mass marker, were also assessed. In patient cells, our results showed a significant decrease in the expression levels of all observed proteins, including VDAC, indicating a reduction in mitochondrial mass. Notably, treatment with polydatin and nicotinamide resulted in a significant increase in patient fibroblasts for all analyzed proteins (Figure 3). However, upon normalization of mitochondrial protein expression levels to VDAC (mitochondrial mass), we observed nuanced outcomes. For untreated cells from P1 and P2, there was a reduction in the expression levels of all proteins, except SDHB, with a higher decrease observed in the mutant protein (EF-G1) and the proteins encoded by the mitochondrial genome, Mt-ND3, and MtCO2. However, when P1 and P2 fibroblasts were treated with polydatin and nicotinamide, an increase in protein expression levels was observed for all analyzed proteins, even after normalization to mitochondrial mass (Figure S2). Conversely, in the case of untreated P3 cells, normalization by mitochondrial mass revealed an increase in the expression levels of NDUFS1 and ATP5F1A, while the remaining proteins exhibited a decrease, particularly the mutant protein and those encoded by the mitochondrial genome. Nevertheless, the increase in protein expression levels of all analyzed proteins after the treatment was also observed (Figure S3).

**Figure 4:**
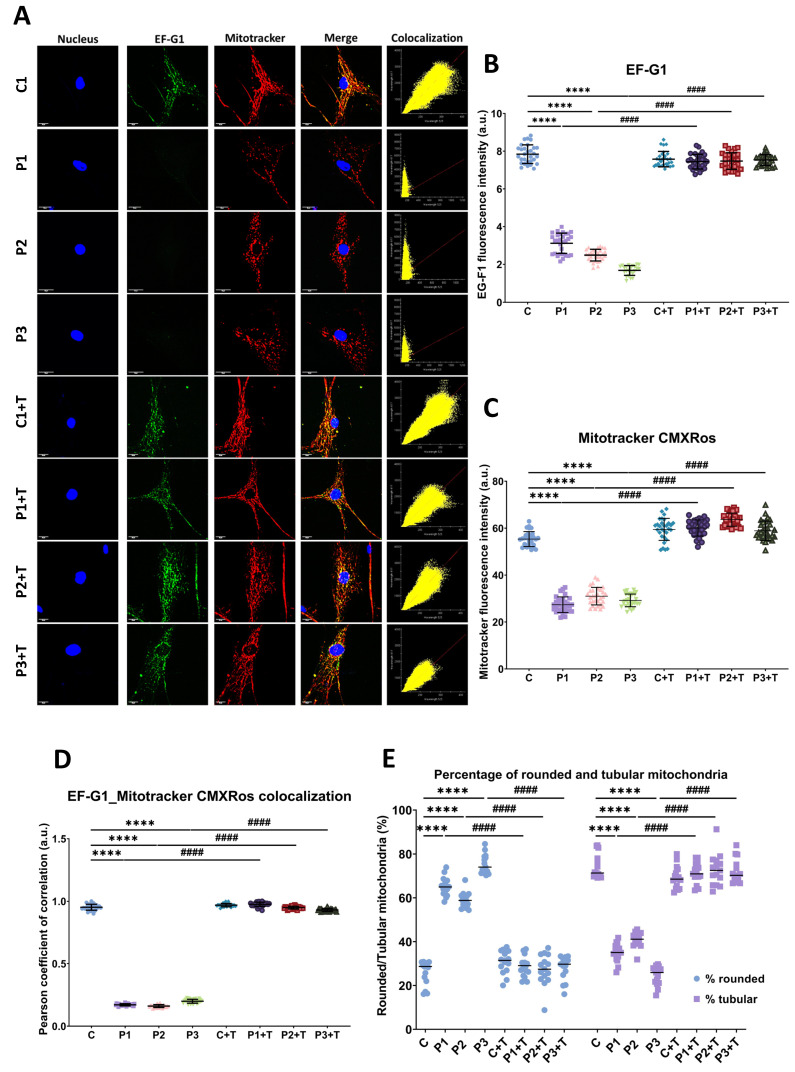
EF-G1 expression and mitochondrial network morphology and polarization by immunofluorescence. Cells were subjected to treatment with polydatin and nicotinamide at 10 µM for seven days (+T). Following this, control (C1) and mutant (P1, P2, P3) cells were incubated with Mitotracker^TM^ CMXRos 100 nM for 45 min, after which they were fixed and immunostained with EF-G1 antibody, while nuclei were visualized by DAPI staining. (**A**) Representative images of untreated and treated fibroblasts, acquired from a DeltaVision microscope. (**B**) Quantification of fluorescence intensity of EF-G1 antibody. (**C**) Quantification of fluorescence intensity of Mitotracker^TM^ CMXRos. (**D**) Pearson coefficient of colocalization between EF-G1 and Mitotracker^TM^ CMXRos. (**E**) Quantification of the percentage of rounded (blue data)/tubular (purple data) mitochondria. Rounded mitochondria were defined as 0.2–0.5 μm^2^, and tubular mitochondria as >0.5 μm^2^. C represents the mean of C1, C2, and C3 cells. Data represent the mean ± SD of three separate experiments (at least 30 images were taken from each condition and experiment). Scale bar = 20 µm. **** *p* < 0.0001 between control and mutant fibroblasts. ^####^ *p* < 0.0001 between untreated and treated patient fibroblasts. a.u.: arbitrary units.

Subsequently, we assessed immunofluorescence staining of EF-G1 protein. Fibroblasts were co-stained with Mitotracker^TM^ CMXRos, a dye reliant on mitochondrial membrane potential. Notably, we observed a significant decrease in EF-G1 immunofluorescence signals in mutant fibroblasts compared to control cells. Treatment with polydatin and nicotinamide led to a substantial increase in EF-G1 immunofluorescence signals in the mutant cells. In contrast, polydatin and nicotinamide supplementation had no discernible effect on control cells (Figure 4A,B). Moreover, we observed that the colocalization between EF-G1 and Mitotracker^TM^ CMXROS was clear in control cells, while in mutant fibroblasts, it was reduced and reverted with polydatin and nicotinamide supplementation (Figure 4A,D). Furthermore, mutant fibroblasts exhibited a reduction in mitochondrial membrane potential and a disruption of the mitochondrial network, accompanied by an increase in rounded mitochondria, in comparison to control fibroblasts. Importantly, both these alterations—decreased mitochondrial membrane potential and network fragmentation—were effectively reversed with polydatin and nicotinamide supplementation (Figure 4C,E). For the quantification of rounded and tubular mitochondria, cells were stained with Mitotracker^TM^ Deep Red FM which is not dependent on mitochondrial membrane potential (images in Figure S4).

**Figure 5:**
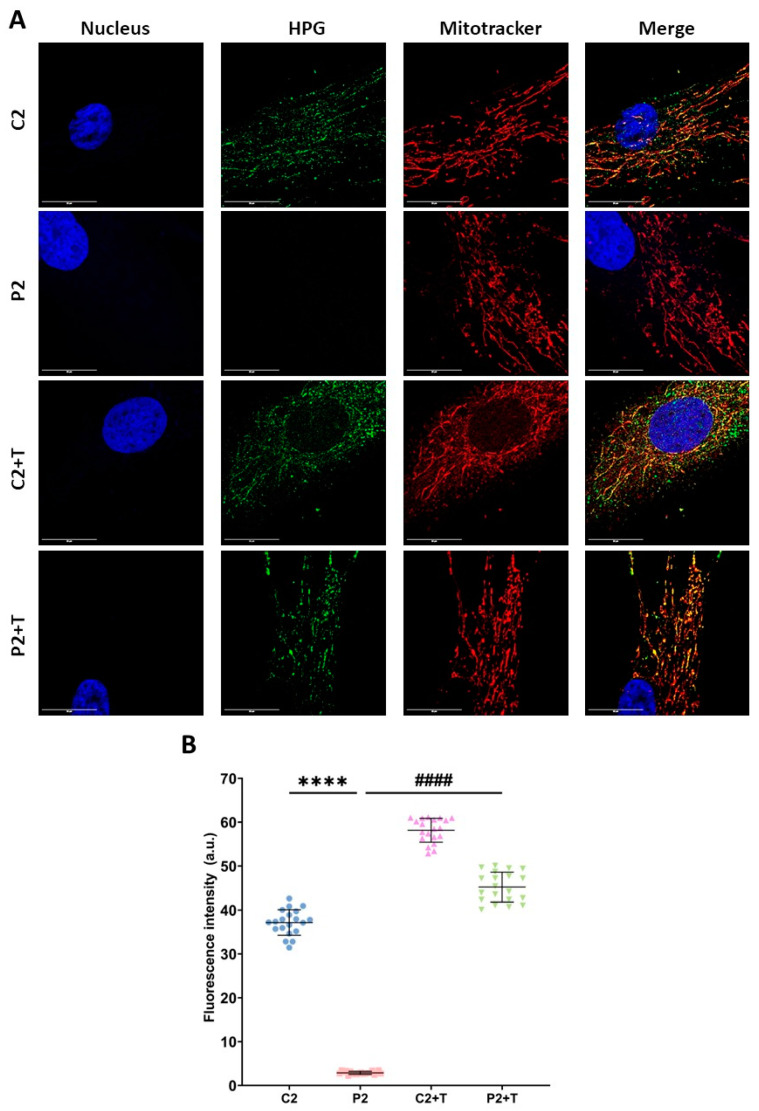
Mitochondrial protein synthesis. Control (C2) and mutant (P2) cells were treated with polydatin and nicotinamide at 10 μM for seven days (+T). The FUNCAT protocol was employed for total protein synthesis. To specifically measure mitochondrial protein synthesis, cells were treated with cycloheximide 50 µg/mL for 20 min to inhibit cytosolic protein synthesis. Subsequently, cells were incubated with Mitotracker^TM^ Deep Red FM for 45 min and then with HPG 488 Alexa Fluor for 30 min. (**A**) Representative images were acquired using a Zeiss880 ‘Airyscan’ microscope. (**B**) Quantification of fluorescence intensity. Data represent the mean ± SD of three separate experiments (at least 30 images were taken from each condition and experiment). **** p < 0.0001 between control and mutant fibroblasts. ^####^ *p* < 0.0001 between untreated and treated patient cells. a.u.: arbitrary units. Scale bar = 20 µm. Refer to additional file: Figure S5 for total and cytosolic protein synthesis; Figure S6 for negative control.

To confirm the deficiency in mitochondrial protein synthesis in mutant fibroblasts, we employed the fluorescent noncanonical amino acid tagging (FUNCAT) protocol to measure total, cytosolic, and mitochondrial protein synthesis in P2 fibroblasts. Consistent with expectations, mitochondrial protein synthesis was significantly diminished in P2 mutant cells. Intriguingly, P2 fibroblasts exhibited a noteworthy increase in mitochondrial protein synthesis upon supplementation with polydatin and nicotinamide (Figure 5).

Taken together, these results suggest that treatment with polydatin and nicotinamide could increase the transcription of the *GFM1* gene and the expression of the mutant protein and other mitochondrial proteins encoded by both nuclear and mitochondrial genomes. Additionally, this treatment could reverse mitochondrial membrane depolarization and fragmentation while also enhancing mitochondrial protein synthesis.

### 3.3. Polydatin and Nicotinamide Supplementation Improved Cell Bioenergetics and Respiratory Complex Activity of Mutant GFM1 Fibroblasts

**Figure 6:**
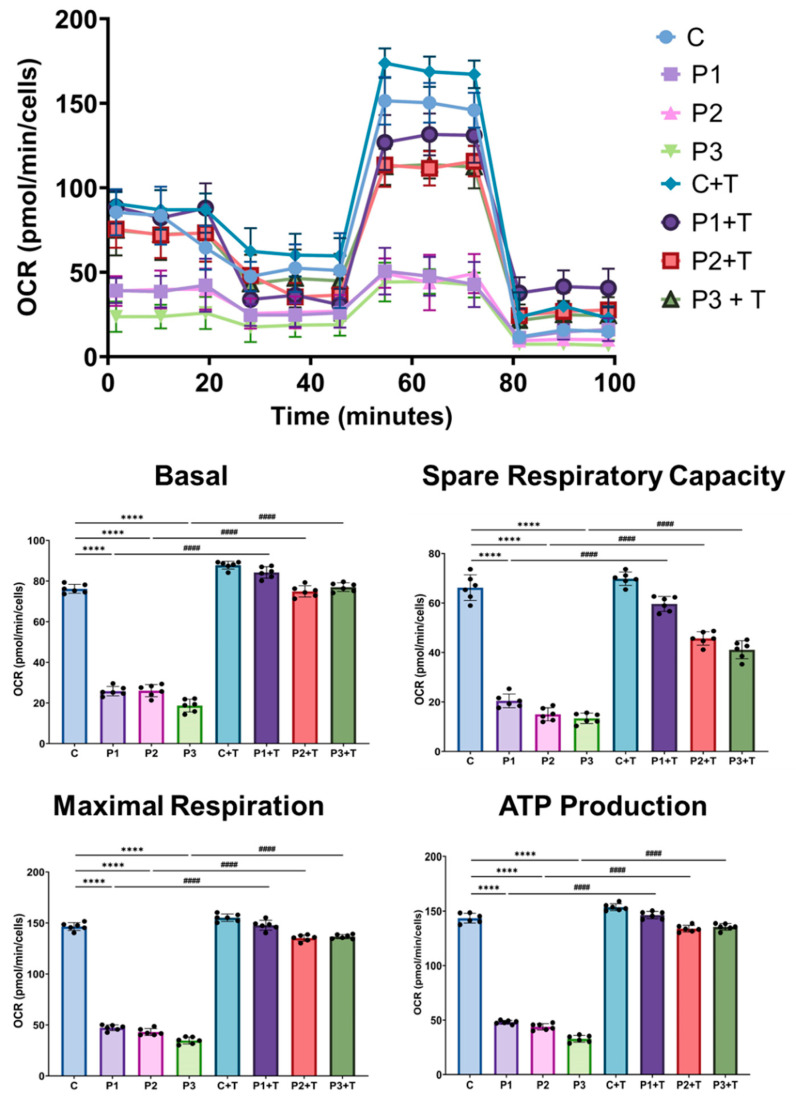
Effect of polydatin and nicotinamide treatment on the Mitostress bioenergetic assay in untreated and treated control (C) and mutant fibroblasts (P1, P2, P3). The mitochondrial respiration profile was measured using the Seahorse XFe24 analyzer. Fibroblasts were treated with polydatin and nicotinamide at 10 µM for seven days (+T). C represents the mean of C1, C2, and C3 cells. Data represent the mean ± SEM of three separate experiments. **** *p* < 0.0001 between control and patient fibroblasts. ^####^ *p* < 0.0001 between untreated and treated mutant *GFM1* cells. OCR: oxygen consumption rate.

Subsequently, we evaluated the impact of the treatment on the bioenergetic profile of mitochondria in mutant cells. For this purpose, we conducted a Mitostress test assay using an XFe24 extracellular flux analyzer (Seahorse Bioscience, Billerica, MA, USA) in both untreated and treated control and patient fibroblasts. Mutant *GFM1* fibroblasts exhibited a significant reduction in basal respiration, maximal respiration, ATP production, and spare respiratory capacity compared to control cells. Remarkably, polydatin and nicotinamide supplementation significantly improved these parameters in all three patient lines (Figure 6).

**Figure 7:**
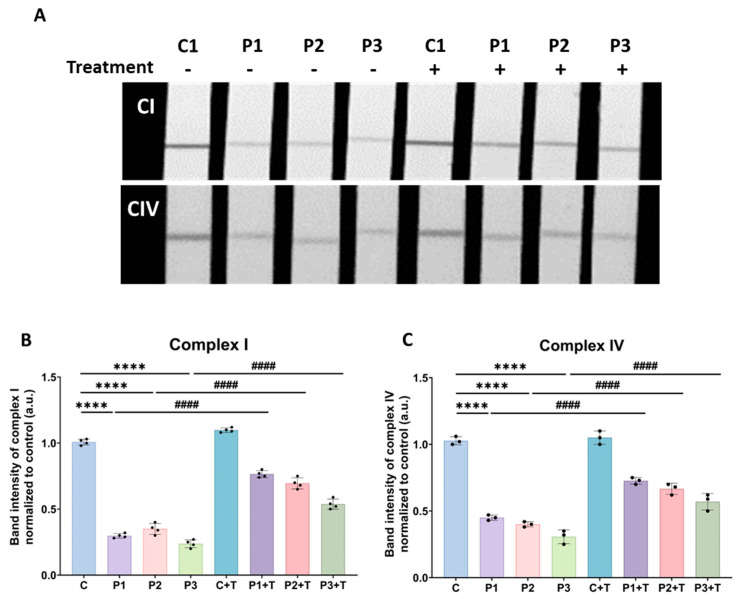
Effect of polydatin and nicotinamide treatment on complex I and complex IV activities in untreated and treated control (C1) and mutant fibroblasts (P1, P2, P3). The treatment with polydatin and nicotinamide at 10 µM was administered for seven days (+T). (**A**) Complex I activity was measured using the Complex I Enzyme Activity Dipstick Assay Kit from Abcam (ab109720). Complex IV activity was measured using the Complex IV Enzyme Activity Dipstick Assay Kit from Abcam (ab109876). (**B**) Band intensity of complex I activity was obtained using ImageLab software. (**C**) Band intensity of complex IV activity was obtained using ImageLab software. C represents the mean of C1, C2, and C3 cells. Data represent the mean ± SEM of three separate experiments. **** *p* < 0.0001 between control and *GFM1* fibroblasts. ^####^ *p* < 0.0001 between untreated and treated patient cells. a.u.: arbitrary units.

In addition, we measured the activity of mitochondrial complexes I and IV, the most affected in *GFM1* mutations [29], using a dipstick assay. In line with mitochondrial dysfunction, complex I and IV activities were diminished in mutant cells. Interestingly, the activity of both mitochondrial complexes exhibited a significant increase following polydatin and nicotinamide supplementation in patient fibroblasts compared to untreated conditions (Figure 7).

The increase in Mitostress assay parameters, as well as in the activity of mitochondrial complexes I and IV, would indicate that the mitochondrial proteins whose expression levels increase with polydatin and nicotinamide treatment would also be functional.

### 3.4. Polydatin and Nicotinamide Supplementation-Activated mtUPR and Mitochondrial Biogenesis

Given that nicotinamide and polydatin could be considered mtUPR activators, we assessed the protein expression levels of mtUPR-associated proteins in untreated and treated control and mutant cells.

Three axes of the mtUPR have been described [30]: the transcriptional canonical mtUPR, where ATF4, ATF5, and C/EBP Homologous Protein (CHOP) are the primary effectors; the SIRT3 mtUPR axis, with SIRT3 as the triggering factor; and, thirdly, the intermembrane space mtUPR, which is activated only when mitochondrial stressors are in the mitochondrial intermembrane space, inducing mitochondrial biogenesis via Nrf1 on the one hand and OMI protease on the other [15]. In this work, we focused on the first and second axes since the third is more specific and occurs only when mitochondrial stress is confined to the intermembrane space, whereas in this case, there is stress throughout the mitochondria.

**Figure 8:**
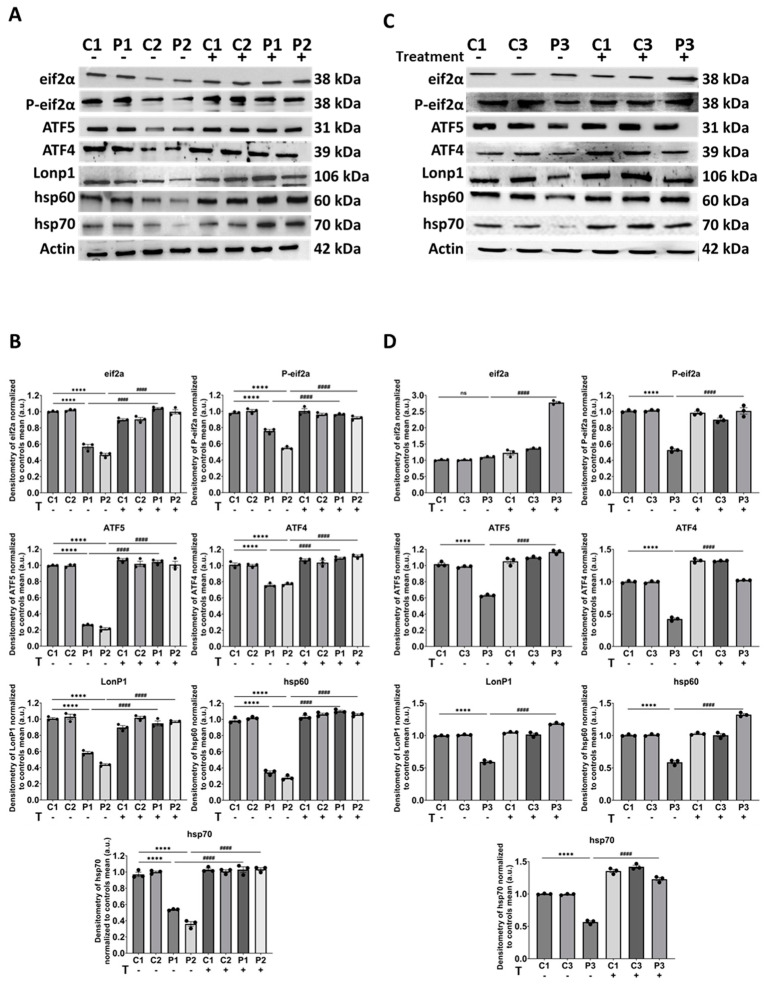
Expression levels of transcriptional canonical mtUPR proteins in control and mutant cells with and without treatment. Polydatin and nicotinamide at 10 µM were used for seven days. (**A**) Immunoblotting analysis of transcriptional canonical mtUPR-associated proteins in control (C1, C2) and mutant (P1, P2) fibroblasts. Actin was used as the loading control. (**B**) Band densitometry of Western blot data referred to actin and normalized to the mean of controls. (**C**) Immunoblotting analysis of transcriptional canonical mtUPR-associated proteins in control (C1, C3) and mutant (P3) cells. Actin was used as the loading control. Original images can be found in Supplementary Materials. (**D**) Band densitometry of Western blot data referred to actin and normalized to the mean of controls. Data represent the mean ± SEM of three independent experiments. **** *p* < 0.0001 between control and *GFM1* fibroblasts. ^####^ *p* < 0.0001 between untreated and treated *GFM1* cells. a.u.: arbitrary units. ns: not significant.

We performed Western blot analysis targeting eif2α and its phosphorylated active form (P-eif2α) as initiators of the transcriptional canonical mtUPR and the Integrated Stress Response (ISR) [31]. ATF4 and ATF5, as the primary triggers of this pathway [32], and mitochondrial chaperones Hsp60 and Hsp70, along with mitochondrial protease Lonp1 [33], were also included. The expression levels of transcriptional canonical mtUPR-related proteins were significantly reduced in mutant cells, and this reduction was at least partially corrected by polydatin and nicotinamide treatment (Figure 8)**.**

**Figure 9:**
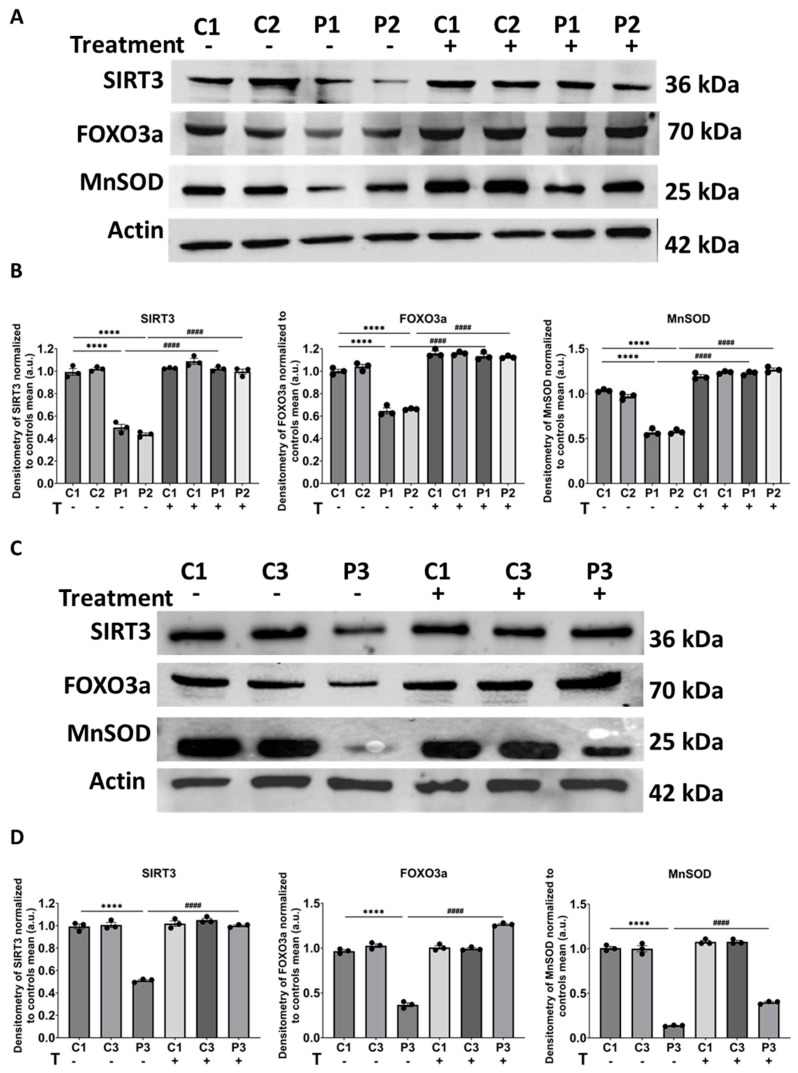
Expression levels of SIRT3 mtUPR proteins in control and mutant cells with and without treatment. Polydatin and nicotinamide at 10 µM were used for seven days. (**A**) Immunoblotting analysis of SIRT3 mtUPR-associated proteins in control (C1, C2) and mutant (P1, P2) cells. Actin was used as the loading control. (**B**) Band densitometry of Western blot data referred to actin and normalized to the mean of controls. (**C**) Immunoblotting analysis of SIRT3 mtUPR-associated proteins in control (C1, C3) and mutant (P3) cells. Actin was used as the loading control. Original images can be found in Supplementary Materials. (**D**) Band densitometry of Western blot data referred to actin and normalized to the mean of controls. Data represent the mean ± SEM of three independent experiments. **** *p* < 0.0001 between control and *GFM1* fibroblasts. ^####^ *p* < 0.0001 between untreated and treated *GFM1* cells. a.u.: arbitrary units.

Moreover, we examined the protein expression levels of SIRT3 mtUPR-related proteins including SIRT3, FOXO3a, and MnSOD as a key antioxidant enzyme in this stress response axis [34,35]. As expected, the expression levels of SIRT3 mtUPR-associated proteins were reduced in mutant cells compared to control fibroblasts. Notably, we observed a significant increase in *GFM1* cells treated with polydatin and nicotinamide (Figure 9).

Therefore, polydatin and nicotinamide treatment would be activating the transcriptional canonical and antioxidant axes of the mtUPR.

**Figure 10:**
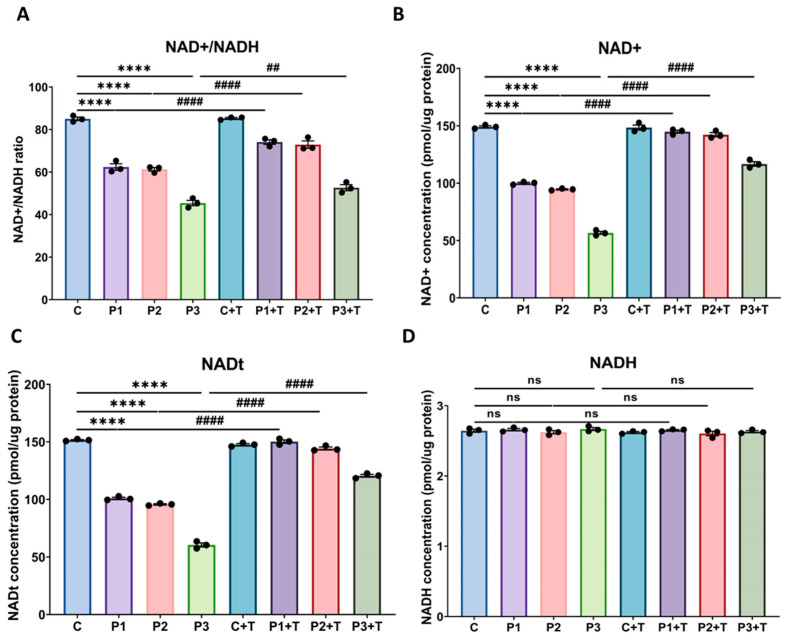
Effect of polydatin and nicotinamide supplementation on cellular NAD^+^/NADH ratio, NAD^+^, NADH, and NADt levels in both untreated and treated control (**C**) and mutant cells (P1, P2, P3). Cells were treated with polydatin and nicotinamide at 10 µM for seven days (+T). The assay was performed using the NAD^+^/NADH Assay Kit (Colorimetric) from Abcam (ab221821). (**A**) NAD^+^/NADH ratio. (**B**) NAD^+^ concentration (quantified by subtracting NADH from NADt). (**C**) NADt concentration (NAD^+^ and NADH total content). (**D**) NADH concentration. C represents the mean of C1, C2, and C3 cells. Data represent the mean ± SEM of three separate experiments. **** *p* < 0.0001 between control and patient fibroblasts. ^##^ *p* < 0.01, *^####^ p* < 0.0001 between untreated and treated mutant *GFM1* cells. ns: not significant.

Additionally, given the interconnection between the mtUPR and NAD^+^ levels, we quantified total NAD (NADt), NAD^+^, and NADH levels, along with the NAD^+^/NADH ratio. Our findings revealed a noteworthy reduction in the NAD^+^/NADH ratio (Figure 10A), as well as NAD^+^ (Figure 10B) and NADt levels (Figure 10C), in mutant cells in comparison to control fibroblasts. Conversely, no significant differences were observed in NADH levels between control and patient cells (Figure 10D). Importantly, nicotinamide and polydatin supplementation significantly elevated NAD^+^ and NADt levels, as well as the NAD^+^/NADH ratio, in mutant P1, P2, and P3 cells (Figure 10A–C).

**Figure 11:**
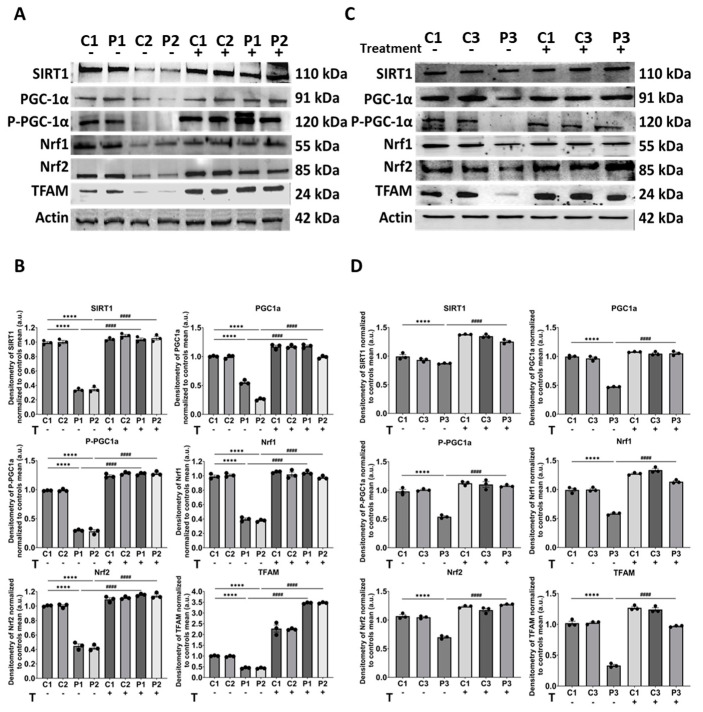
Expression levels of mitochondrial biogenesis proteins in control and mutant cells with and without treatment. Polydatin and nicotinamide at 10 µM were used for seven days. (**A**) Immunoblotting analysis of mitochondrial biogenesis proteins in control (C1, C2) and mutant (P1, P2) cells. Actin was used as the loading control. (**B**) Band densitometry of Western blot data referred to actin and normalized to the mean of controls. (**C**) Immunoblotting analysis of mitochondrial biogenesis proteins in control (C1, C3) and mutant (P3) fibroblasts. Actin was used as the loading control. Original images can be found in Supplementary Materials. (**D**) Band densitometry of Western blot data referred to actin and normalized to the mean of controls. Data represent the mean ± SEM of three independent experiments. **** *p* < 0.0001 between control and *GFM1* fibroblasts. ^####^ *p* < 0.0001 between untreated and treated *GFM1* cells. a.u.: arbitrary units.

Moreover, we conducted Western blot analysis of proteins involved in mitochondrial biogenesis, such as SIRT1, PGC1α and its phosphorylated active form (P-PGC1α), Nrf1, Nrf2, and TFAM. Patient cells showed low protein expression levels, which were partially corrected by nicotinamide and polydatin supplementation, suggesting the activation of mitochondrial biogenesis (Figure 11).

**Figure 12:**
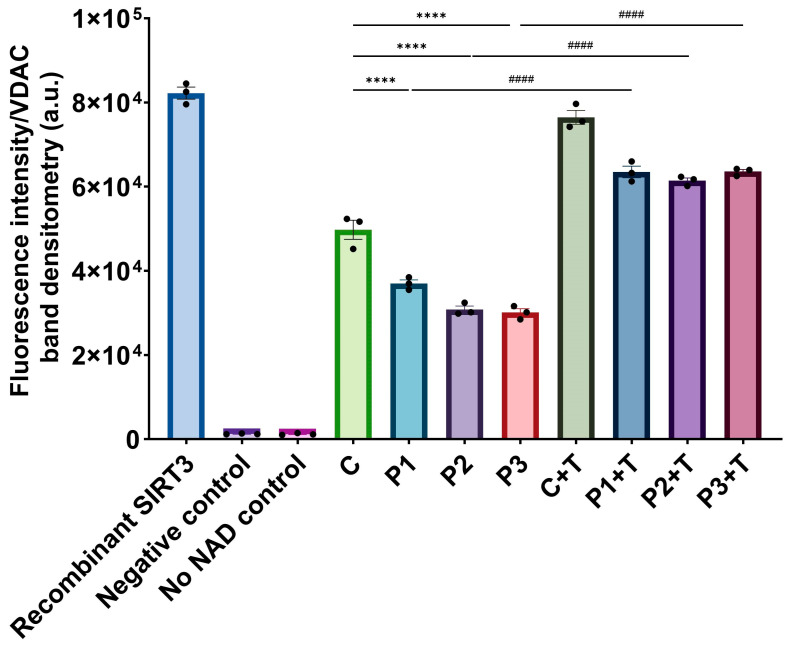
Effect of polydatin and nicotinamide treatment for seven days (+T) on SIRT3 activity in the mitochondrial fraction of untreated and treated control (C) and mutant *GFM1* fibroblasts (P1, P2, P3). SIRT3 activity was measured using the SIRT3 Activity Assay Kit from Abcam (ab156067). Pure SIRT3 was used as a positive control, and no enzyme and no NAD were used as negative controls. Fluorescence was measured by a POLARstar Omega plate reader. C represents the mean of C1, C2, and C3 cells. Data represent the mean ± SEM of three independent experiments. Results of SIRT3 activity in the mitochondrial fractions were divided between the band densitometry of VDAC for normalization. **** p < 0.0001 between control and patient fibroblasts. ^####^ *p* < 0.0001 between untreated and treated mutant *GFM1* fibroblasts. a.u.: arbitrary units. Refer to additional file: Figure S7 for purity of cellular fractions.

Next, considering that NAD^+^ is a sirtuin cofactor and SIRT3 protein expression levels increased with polydatin and nicotinamide treatment, we measured the deacetylase activity of SIRT3 in the mitochondrial fraction. For this purpose, we isolated mitochondria and then measured SIRT3 activity. We found a significant decrease in SIRT3 activity in patient mitochondria compared to the control, and this decrease was reversed with polydatin and nicotinamide supplementation (Figure 12). We also assessed the purity of cellular fractions by Western blot (Figure S7).

**Figure 13:**
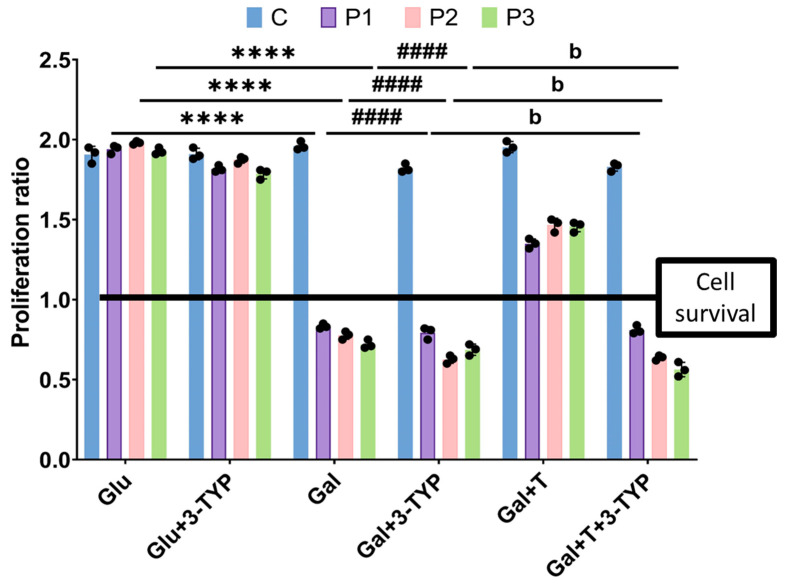
Quantification of the cellular proliferation ratio of galactose screening with 3-TYP. 3-TYP at 32 nM was used as a SIRT3 inhibitor. Control (C) and mutant (P1, P2, P3) cells were initially seeded in glucose medium and treated either with polydatin and nicotinamide at 10 μM alone (T) or T along with 3-TYP at 32 nM. After 3 days, the glucose medium was changed to galactose, T and 3-TYP were refreshed, and cell counting was obtained immediately (T0) and 72 h later (T72) using the BioTek Cytation 1. The proliferation ratio was calculated by dividing the number of cells at T72 by the number of cells at T0. Results equal to 1 mean cell survival, higher than 1 cell proliferation, and under than 1 cell death. C represents the mean of C1, C2, and C3 cells. Data represent the mean ± SEM of three independent experiments. **** *p* < 0.0001 between glucose and galactose medium. ^####^ *p* < 0.0001 between cells in galactose and galactose medium with the treatment. ^b^ *p* < 0.0001 between cells in galactose medium with the treatment and with or without 3-TYP. Glu: glucose. Gal: galactose. Refer to additional file: Figure S8 for representative images of galactose screening with 3-TYP.

To further validate the impact of SIRT3 activation through nicotinamide and polydatin supplementation, we conducted a replication of the galactose screening assay using a specific SIRT3 inhibitor, 3-TYP. Both control and patient fibroblasts were exposed to 3-TYP at 32 nM for three days in glucose and galactose media, with or without the treatment. Control cells exhibited consistent growth rates in both glucose and galactose media, regardless of treatment or the presence of 3-TYP. However, mutant cells died in galactose medium and galactose medium with 3-TYP. Notably, they also failed to survive in galactose medium even with polydatin and nicotinamide supplementation when the SIRT3 inhibitor was present (Figure 13). These findings suggest a pivotal role for SIRT3 in the mechanism of action of polydatin and nicotinamide.

**Figure 14:**
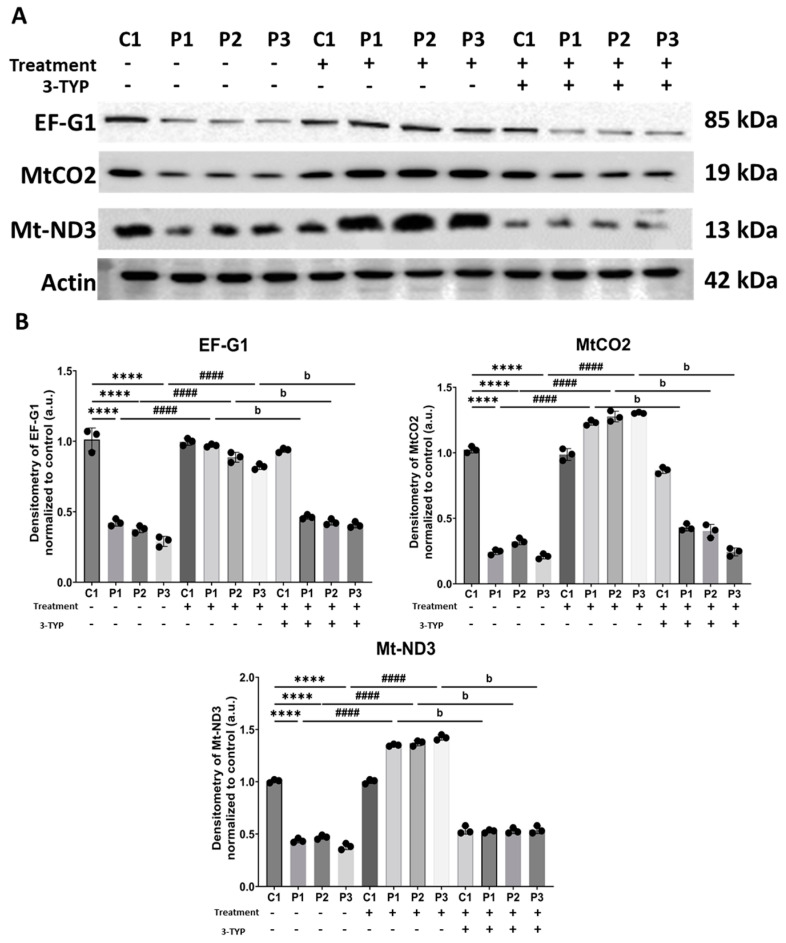
Expression levels of EF-G1, MtCO2, and Mt-ND3 in control (C1) and mutant fibroblasts (P1, P2, P3). Cells were treated with nicotinamide and polydatin, and with nicotinamide and polydatin along with 3-TYP for seven days. (**A**) Western blot analysis of the mutant protein (EF-G1) and two mitochondrial proteins encoded by mtDNA (MtCO2 and Mt-ND3). Actin was used as the loading control. Original images can be found in Supplementary Materials. (**B**) Band densitometry of Western blot data referred to actin and normalized to the control. Data represent the mean ± SEM of three separate experiments. **** *p* < 0.0001 between control and mutant *GFM1* cells. ^####^ *p* < 0.0001 between untreated and treated patient cells. ^b^ *p* < 0.0001 between patient cells treated with nicotinamide and polydatin alone or treatment along with 3-TYP. a.u.: arbitrary units.

The target of our treatment, SIRT3, was confirmed through a Western blot analysis of the mutant protein (EF-G1) and two proteins encoded by the mitochondrial genome (MtCO2 and Mt-ND3). The analysis was conducted in the presence of the treatment alone and in combination with the specific SIRT3 inhibitor, 3-TYP. All three observed proteins exhibited an increase in their expression levels with the treatment, which was not achievable in the presence of 3-TYP (Figure 14). This substantiates that SIRT3 is indeed the target of polydatin and nicotinamide supplementation.

### 3.5. Polydatin and Nicotinamide Supplementation Increased EF-G1 and MtCO2 Relative Fluorescence Intensity in Induced Neurons

While patient-derived fibroblast models provide valuable insights into the pathophysiology of mitochondrial diseases and potential therapeutic interventions, it is essential to recognize that the most affected cells in these diseases are neurons and muscle cells. To overcome this limitation, both control and P2-derived fibroblasts were transdifferentiated into induced neurons (iNs) using lentiviral vectors containing proneural-specific genes [24].

Thirty days after the infection, cells exhibited immunoreactivity against Tau, a microtubule-associated protein found in neuronal axons. Conversion efficiency and neuronal purity were calculated based on Tau^+^ cells, resulting in a conversion efficiency of 19.57 ± 2.13% in control cells and 18.88 ± 3.54% in mutant cells, while neuronal purity was 74.62 ± 2.71% in control cells and 76.63 ± 2.34% in mutant cells (Figure S9).

**Figure 15:**
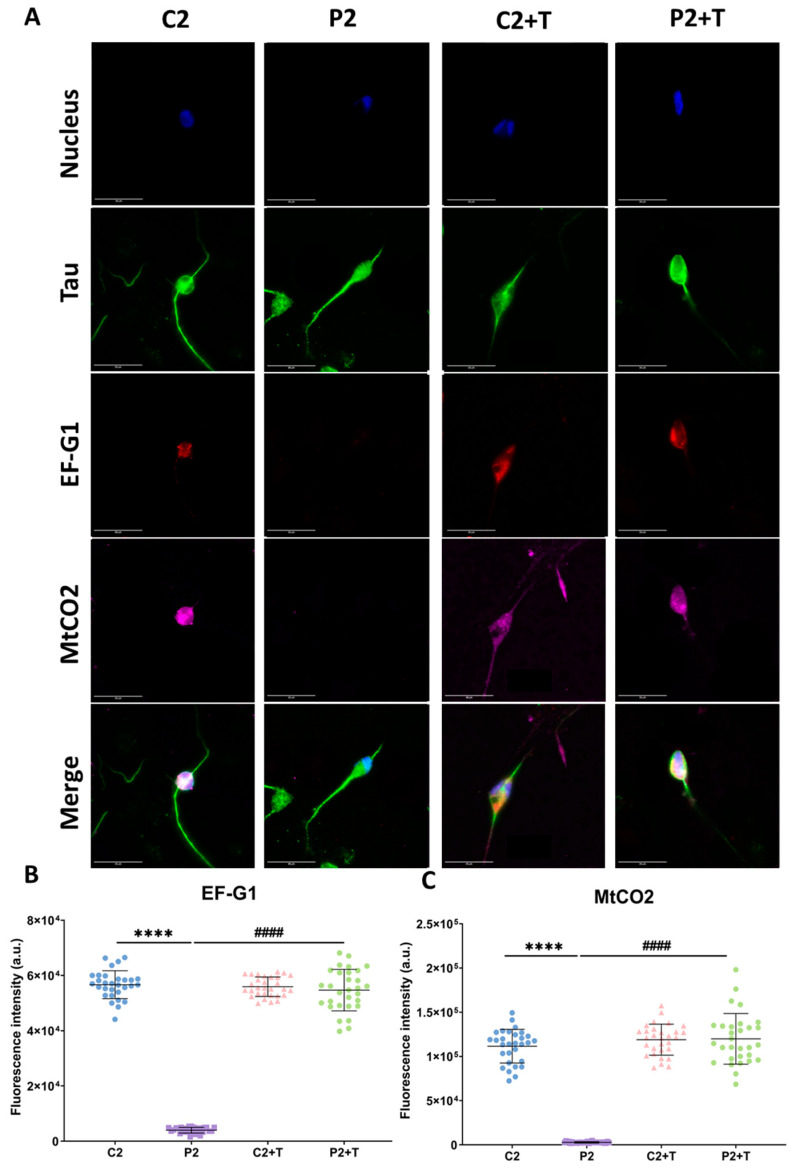
Immunofluorescence analysis of EF-G1 and MtCO2 in iNs. Control (C2) and mutant (P2) cells, reprogrammed from fibroblasts to iNs, were subjected to treatment with polydatin and nicotinamide at 10 µM for seven days (+T). Following this, both control and mutant cells were fixed and immunostained using antibodies against Tau, EF-G1, and MtCO2, while nuclei were visualized by DAPI staining. (**A**) Representative images of untreated and treated control and mutant iNs, acquired from a Zeiss880 ‘Airyscan’ microscope. (**B**) Quantification of fluorescence intensity of EF-G1 antibody. (**C**) Quantification of fluorescence intensity of MtCO2 antibody. Data represent the mean ± SD of three separate experiments (at least 30 images were taken from each condition and experiment). Scale bar = 20 µm. **** *p* < 0.0001 between control and mutant fibroblasts. ^####^ *p* < 0.0001 between untreated and treated patient fibroblasts. a.u.: arbitrary units.

Next, we evaluated the efficacy of polydatin and nicotinamide supplementation in these mutant *GFM1* iNs by performing immunofluorescence staining of EF-G1 and MtCO2 to provide a semiquantitative assessment of protein expression. Tau protein was used as a neuronal marker. In mutant iNs, EF-G1 and MtCO2 immunofluorescence signals were nearly absent in comparison to control cells. Interestingly, treatment with polydatin and nicotinamide significantly increased the immunofluorescence intensity of both proteins in mutant iNs, as previously observed in fibroblasts (Figure 15), suggesting that polydatin and nicotinamide treatment would also be effective in neurons, one of the most affected cell types in mitochondrial diseases.

## 4. Discussion

The treatment of mitochondrial diseases is challenging as there are no curative therapeutic options available for the majority of them [36]. Currently, various strategies are employed for the treatment of mitochondrial diseases. Symptomatic treatments are commonly used, while specific treatments targeting certain diseases include the use of thiamine, riboflavin, biotin, or niacin for cofactor deficiency mitochondrial diseases [37], and idebenone for LHON patients [38]. In addition, approved treatments such as L-arginine and taurine are used to prevent stroke-like episodes in MELAS [39,40]. Another strategy involves increasing mitochondrial content in cells, utilizing agents like acipimox [41], bezafibrate [42], or omaveloxone [43]. Nevertheless, the safety and efficacy of these treatments remain unclear [43,44,45]. Protecting mitochondria from damage is also considered a potential treatment strategy, with pharmacological agents such as cysteamine [46], elamipretide [47], or EPI-743 [48] targeting antioxidant production and reducing ROS. However, more clinical trials are necessary to confirm their efficacy. In addition, dichloroacetate, a pharmacological agent that improves the efficiency of OXPHOS, is currently in clinical trials [49]. Moreover, dietary supplements have been also used for the treatment of mitochondrial diseases since they could increase ATP production, remove ROS from cells, or bypass cellular defects [50,51,52,53,54,55,56,57,58]. Notwithstanding, despite the large number of studies on the beneficial effect of dietary supplements, there is no robust evidence to support their use in the treatment of these conditions.

On the other hand, gene therapy has emerged as a promising treatment for mitochondrial diseases and a potential cure for these life-limiting disorders. However, only one gene therapy for a specific mitochondrial disease, LHON, is currently in a clinical trial [59,60]. As a result, this therapeutic approach is still a long way from becoming standard clinical practice.

However, the number of patients diagnosed with these pathologies is steadily increasing, thanks to recent advances in next-generation sequencing techniques [61].

Fibroblasts serve as a robust model for the study of numerous diseases, as evidenced by numerous studies conducted over the years [26,62,63,64,65,66,67,68,69,70]. In this work, we establish that fibroblasts derived from patients with mutations in the *GFM1* gene constitute a valuable experimental model for investigating mitochondrial diseases. These cells exhibit several typical pathological manifestations of mitochondrial dysfunction, including susceptibility to death in galactose medium, reduced expression and synthesis of mitochondrial proteins, and perturbed cellular bioenergetics.

As far back as 1992, Robinson et al. [28] observed that a switch from glucose to galactose in mitochondrial mutant fibroblasts resulted in inhibited cell growth, and the survival of mutant cells was contingent on the extent of the OXPHOS defect. Culturing mitochondrial mutant cells in galactose medium has also been used to increase sensitivity to mitochondrial toxins in drug screening studies [71,72,73]. In addition, other researchers have used galactose screening to identify compounds capable of inducing metabolic shifts [74].

In our study, the fact that *GFM1* mutant fibroblasts died in galactose medium provided us with an extremely useful tool for screening pharmacological compounds with therapeutic potential.

mtUPR serves as a mitochondrial compensatory mechanism, primarily aimed at preserving or repairing damaged mitochondria [75]. For that purpose, when there is mitochondrial stress, mtUPR activates a transcriptional response consisting of the activation of mitochondrial chaperones and proteases. These components play crucial roles in either folding misfolded or unfolded proteins or degrading aberrant proteins. Importantly, mtUPR also triggers the mitochondrial antioxidant system, thereby reducing ROS overproduction [76]. This multifaceted response enhances the recovery of mitochondrial function without necessitating mitophagy.

Since the description of this stress response, three mtUPR axes have been described [30]. The first axis is the transcriptional canonical mtUPR, which promotes the activation of molecular chaperones and proteases [16]. The second axis is the SIRT3-mtUPR axis, regulating the expression of antioxidant enzymes such as MnSOD and catalase via the activation of FOXO3a [17]. Lastly, a third axis, the intermembrane space-mtUPR axis, is associated with the accumulation of misfolded and unfolded proteins in the mitochondrial intermembrane space [77]. Given the presence of mitochondrial dysfunction in mitochondrial diseases, it is plausible that all three axes could be activated. However, mitochondrial malfunctioning leads to dysregulation of all these compensatory mechanisms, resulting in the downregulation of mtUPR and, therefore, a failure of this protective response.

Activation of mtUPR has been proposed as a therapeutic target in several diseases [15,78,79], encompassing neurodegenerative [80,81,82,83,84,85,86], cardiovascular [87,88,89,90,91], metabolic [92,93,94], cancer [95,96,97,98,99,100,101,102,103,104,105,106], and mitochondrial diseases [8,14]. Taking advantage of this and the screening system based on cell culture in galactose medium, we tested different activators of mtUPR. Notably, we observed that the combination of nicotinamide and polydatin in a cocktail allowed the survival of mutant fibroblasts under nutritional stress.

Nicotinamide serves as a cofactor in a wide range of metabolic reactions, including the citric acid cycle, OXPHOS, and glycolysis, among others [107]. Recent research has expanded our understanding of nicotinamide, revealing its involvement not only in metabolic reactions but also in crucial cellular processes such as transcription, cellular viability, and DNA repair mechanisms [108]. The supplementation of nicotinamide or its precursors has been a longstanding strategy for the treatment of age-related diseases [109,110,111,112], neurodegenerative disorders [113,114,115,116,117], and cancer [118,119,120,121]. Some authors have proposed that the beneficial effects of nicotinamide in treating diseases stem from the activation of NAD-dependent enzymes, particularly sirtuins [122,123,124]. Sirtuins, as deacetylases, play a key role in multiple cellular processes. In fact, 63% of mitochondrial diseases are subject to an acetylation/deacetylation cycle that is an important regulatory mechanism for mitochondrial function [125]. This mitochondrial deacetylation would be carried out by SIRT3, the main sirtuin with deacetylase activity present in mitochondria [126]. Moreover, SIRT3 serves as the initiator of one of the mtUPR axes, leading to antioxidant activity that protects mitochondria from oxidative stress in the face of damage [127]. Therefore, we suggest that SIRT3 and mtUPR could be two potential therapeutic targets for the treatment of mitochondrial diseases, as we have observed in this study.

On the other hand, polydatin, a resveratrol glucoside, has been used for the treatment of various diseases due to its versatile properties, including anti-inflammatory, hypoglycemic, antipyretic, diuretic, expectorant, hypolipidemic, hypouricemic, anticarcinogenic, antioxidant, and anti-atherosclerotic effects [128,129,130,131,132]. Its antioxidant capability is based on the activation of Nrf2, the most important transcription factor against oxidative stress that is also involved in mtUPR [133]. Moreover, studies indicate that treatment with polydatin results in increased SIRT1 expression, promoting mitochondrial biogenesis and thus, enhancing mitochondrial function [134].

To this end, in our study, we have used a cocktail based on the combination of nicotinamide and polydatin at 10 µM, which has led to significant improvements in the pathophysiology of fibroblasts derived from patients with *GFM1* mutations, increasing the expression and content of mitochondrial proteins, enhancing cellular bioenergetics, elevating the activity of mitochondrial complexes I and IV, and stimulating mitochondrial biogenesis. The slight differences observed in the pathophysiology and treatment response between the cells of the different patients could be due to both the distinct mutations and the genetic backgrounds of each individual.

Furthermore, our findings indicate that the effect of this cocktail is produced through the activation of mtUPR, particularly SIRT3. The addition of a specific inhibitor of this sirtuin caused the death of mutant cells in galactose medium and prevented the increase in the expression levels of three mitochondrial proteins even with treatment supplementation.

Additionally, we have demonstrated in iNs obtained by direct reprogramming that polydatin and nicotinamide supplementation increased EF-G1 and MtCO2 expression levels, suggesting an upregulation of mitochondrial translation in neuronal cells. This observation strengthens the notion that treatment with polydatin and nicotinamide could be a potential therapeutic option for patients with *GFM1* mutations. The treatment exhibited positive effects on key pathophysiological features in both fibroblasts and iNs, emphasizing its potential significance for improving conditions in one of the most affected cell types in mitochondrial diseases.

## 5. Conclusions

In this work, we have established a suitable cellular model for investigating mitochondrial diseases using fibroblasts derived from patients with mutations in the *GFM1* gene. In addition, we have developed a pharmacological screening platform, leveraging the fact that patient fibroblasts struggle to survive in galactose medium. Using this approach, we identified a potential therapeutic cocktail consisting of nicotinamide and polydatin, demonstrating significant improvements in mitochondrial function across three *GFM1* mutant fibroblast cell lines and one iNs cell line obtained by direct reprogramming from patient fibroblasts. Furthermore, we elucidated that the therapeutic mechanism of nicotinamide and polydatin treatment is reliant on the upregulation of mtUPR, mainly through the activation of SIRT3, a mitochondrial deacetylase enzyme.

Our findings propose that nicotinamide and polydatin represent a promising pharmacological therapeutic strategy for patients harboring *GFM1* mutations.

## Data Availability

Data and material are available upon request.

## Supplementary Materials

The following supporting information can be downloaded at: https://www.mdpi.com/article/10.3390/biom14050598/s1, Figure S1: Galactose drug screening; Figure S2: Band densitometry of mitochondrial proteins normalized to the mean of controls and referred to VDAC; Figure S3: Band densitometry of mitochondrial proteins normalized to the mean of controls and referred to VDAC; Figure S4: Images of Mitotracker^TM^ Deep Red FM; Figure S5: Cell protein synthesis; Figure S6: Negative control of cell protein synthesis; Figure S7: Purity of cellular fractions; Figure S8: Effect of SIRT3 inhibition by 3-TYP on galactose screening; Figure S9: iNs generated by direct reprogramming.

## Abbreviations

3-TYP	3-(1H-1,2,3-triazol-4-yl)-pyridine
A	aminoacyl site
*ASCL1*	*Achaete-Scute Family BHLH Transcription Factor 1*
ATF4	Activating Transcription Factor 4
ATP5F1A	ATP synthase F1 subunit alpha
*BRN2*	*POU class 3 homeobox 2*
BSA	Bovine Serum Albumin
CHOP	C/EBP Homologous Protein
COX IV	Cytochrome C Oxidase Subunit IV
DAPI	4′,6-diamidino-2-phenylindole
DMEM	Dulbecco’s Modified Eagle’s Medium
DMSO	Dimethyl Sulfoxide
E	exit site
EF-G1	mitochondrial translation elongation factor G1
eif2α	eukaryotic translation initiation factor 2A
FBS	Fetal Bovine Serum
FCCP	carbonyl cyanide 4-(trifluoromethoxy) phenylhydrazone
FOXO3a	Forkhead box O3
*GFM1*	*G elongation Factor Mitochondrial 1*
HEPES	4-(2-hydroxyethyl)-1-piperazine ethanesulfonic acid
HPG	L-homoproparglyglycine
HRP	horseradish peroxidase
Hsp60	heat shock protein 60
Hsp70	heat shock protein 70
IC_50_	half-maximal inhibitory concentration
iNs	induced neurons
Lonp1	Lon peptidase 1
MnSOD	manganese superoxide dismutase
MtCO2	mitochondrially encoded Cytochrome C Oxidase Subunit II
mtDNA	mitochondrial DNA
mtETC	mitochondrial electron transport chain
Mt-ND3	mitochondrially encoded NADH:Ubiquinone Oxidoreductase Core Subunit 3
mtUPR	mitochondrial Unfolded Protein Response
NAD^+^	nicotinamide adenine dinucleotide
nDNA	nuclear DNA
NDUFS1	NADH:Ubiquinone Oxidoreductase Core Subunit S1
Nrf1	nuclear respiratory factor 1
Nrf2	nuclear respiratory factor 2
OXPHOS	oxidative phosphorylation
P	peptidyl site
PBS	phosphate-buffered saline
PFA	paraformaldehyde
PGC1α	peroxisome proliferator-activated receptor γ co-activator 1α
ROS	reactive oxygen species
SDHB	succinate dehydrogenase complex iron sulfur subunit B
SIRT1	sirtuin 1
SIRT3	sirtuin 3
TFAM	mitochondrial transcription factor A
VDAC	voltage-dependent anion channel
