# Supplementary material for: Polydatin and Nicotinamide Rescue the Cellular Phenotype of Mitochondrial Diseases by Mitochondrial Unfolded Protein Response (mtUPR) Activation
Source: Biomolecules. 2024 May 18;14(5):598. doi: 10.3390/biom14050598 (PMC11118892; doi:10.3390/biom14050598)
Supplement: Supplementary file 1 [file biomolecules-14-00598-s001.zip › biomolecules-2990003-supplementary.pdf]

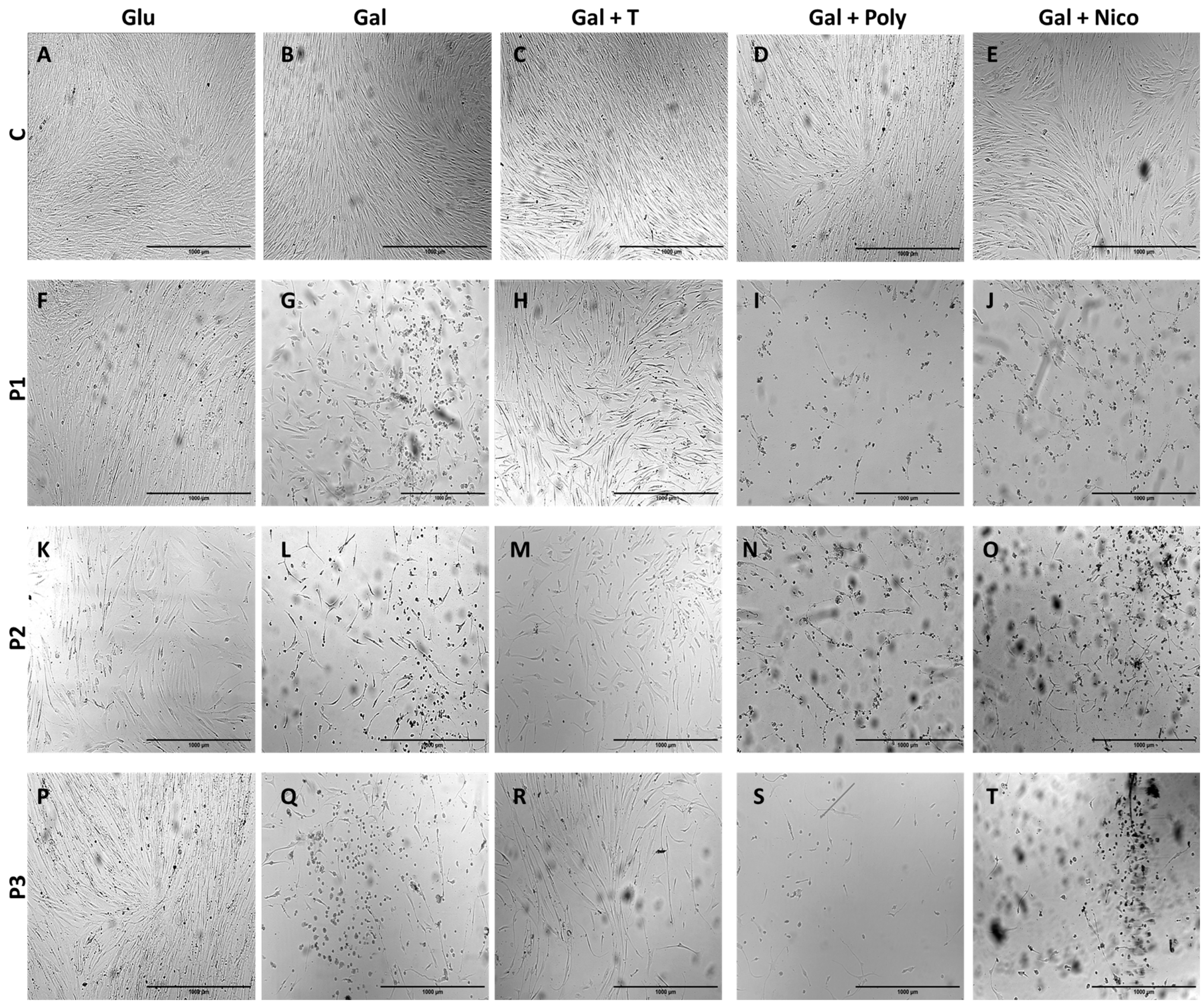

Figure S1. Galactose drug screening. Cells were initially seeded in glucose medium and treated with polydatin and nicotinamide at 10 µM (T), polydatin at 10 µM (Poly) or nicotinamide at 10 µM (Nico). After 3 days, the glucose medium was changed to galactose medium, and treatments were refreshed. In glucose medium, all cell lines grew similarly **(A, F, K, P)**. However, in galactose medium, control cells maintained normal proliferation rate **(B)** while patients' cells experienced cell death **(G, L, Q)**. Polydatin and nicotinamide treatment had no discernible effect on control fibroblasts **(C)** but allowed the survival of mutant cells under stress conditions **(H, M, R).** However, when we treated mutant cells with polydatin individually, they succumbed to cell death **(I, N, S)** and the same occurred when we treated mutant fibroblasts with nicotinamide **(J, O, T).** Images were obtained using the BioTek Cytation 1 Cell Imaging Multi-Mode Reader. Scale bar = 1000 µm. Glu: glucose; Gal: galactose; Poly: polydatin; Nico: Nicotinamide.

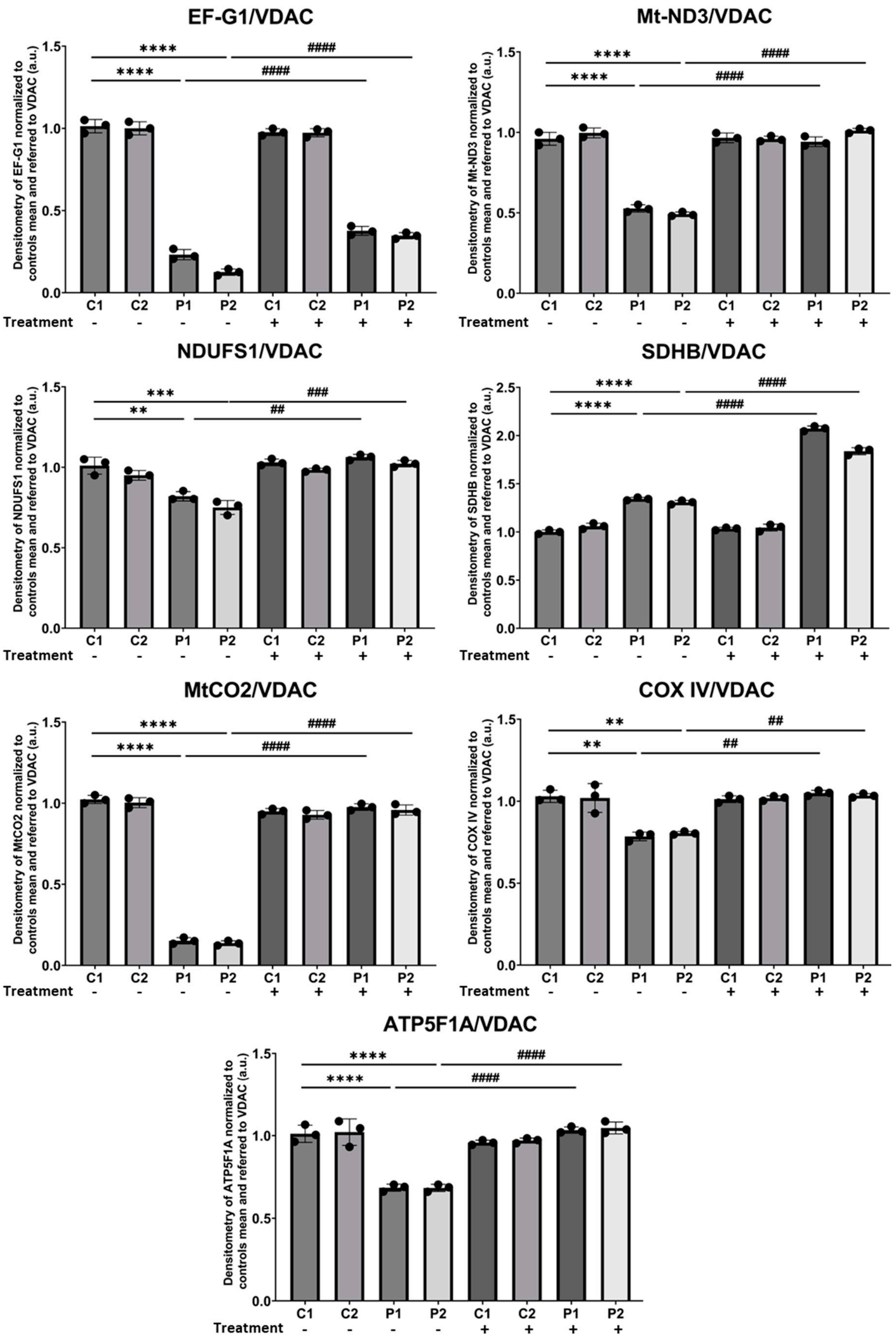

Figure S2. Band densitometry of mitochondrial proteins normalized to the mean of controls and referred to VDAC. Data represent the mean ± SEM of three independent experiments. ****$p<0.0001$ between control and patients' fibroblasts. $^{\#\#\#\#}p<0.0001$ between untreated and treated mutant fibroblasts. a.u.: arbitrary units.

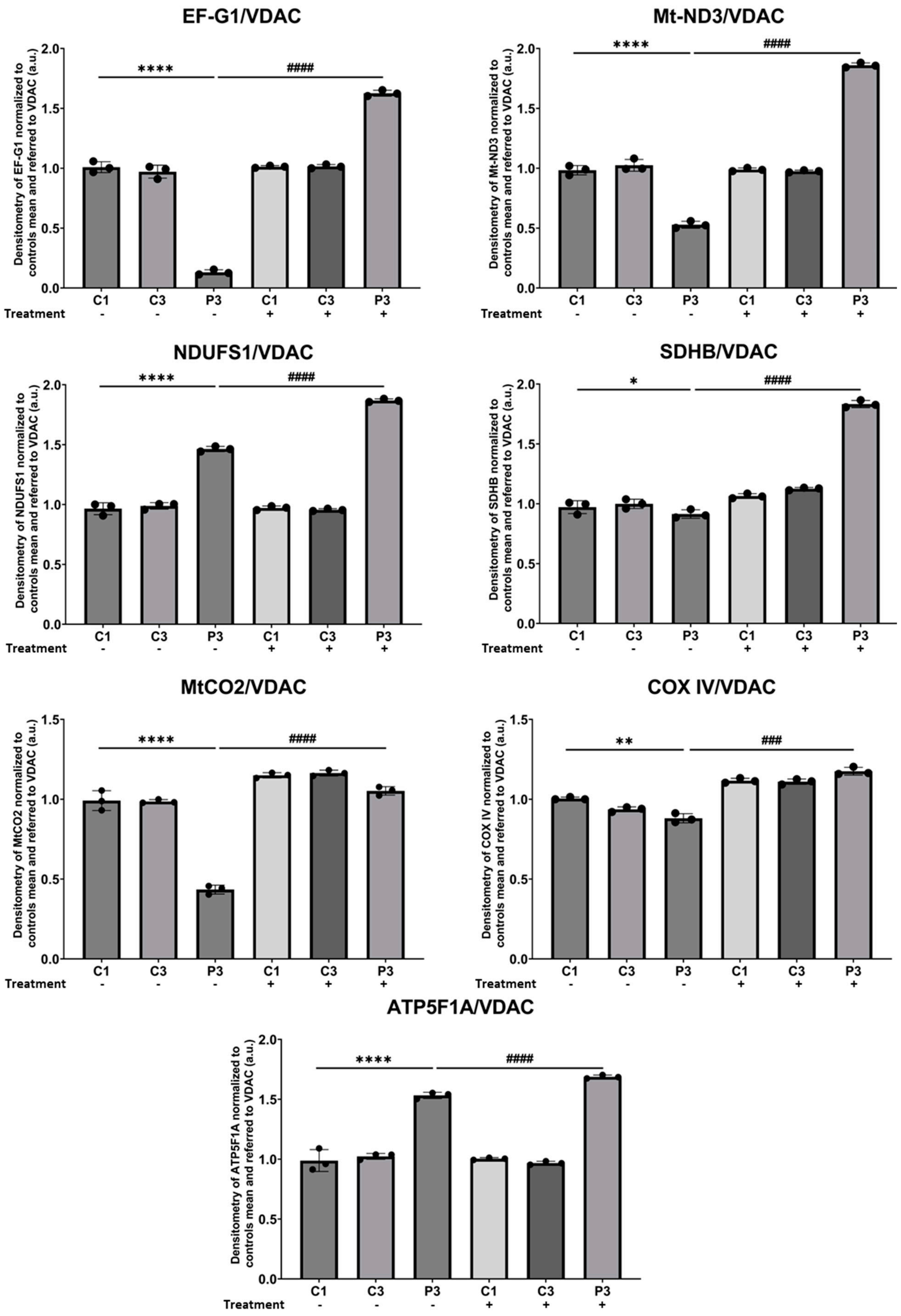

Figure S3. Band densitometry of mitochondrial proteins normalized to the mean of controls and referred to VDAC. Data represent the mean ± SEM of three independent experiments. *$p<0.05$, **$p<0.01$, ****$p<0.0001$ between control and patients' fibroblasts. ###$p<0.001$, ####$p<0.0001$ between untreated and treated mutant fibroblasts. a.u.: arbitrary units.

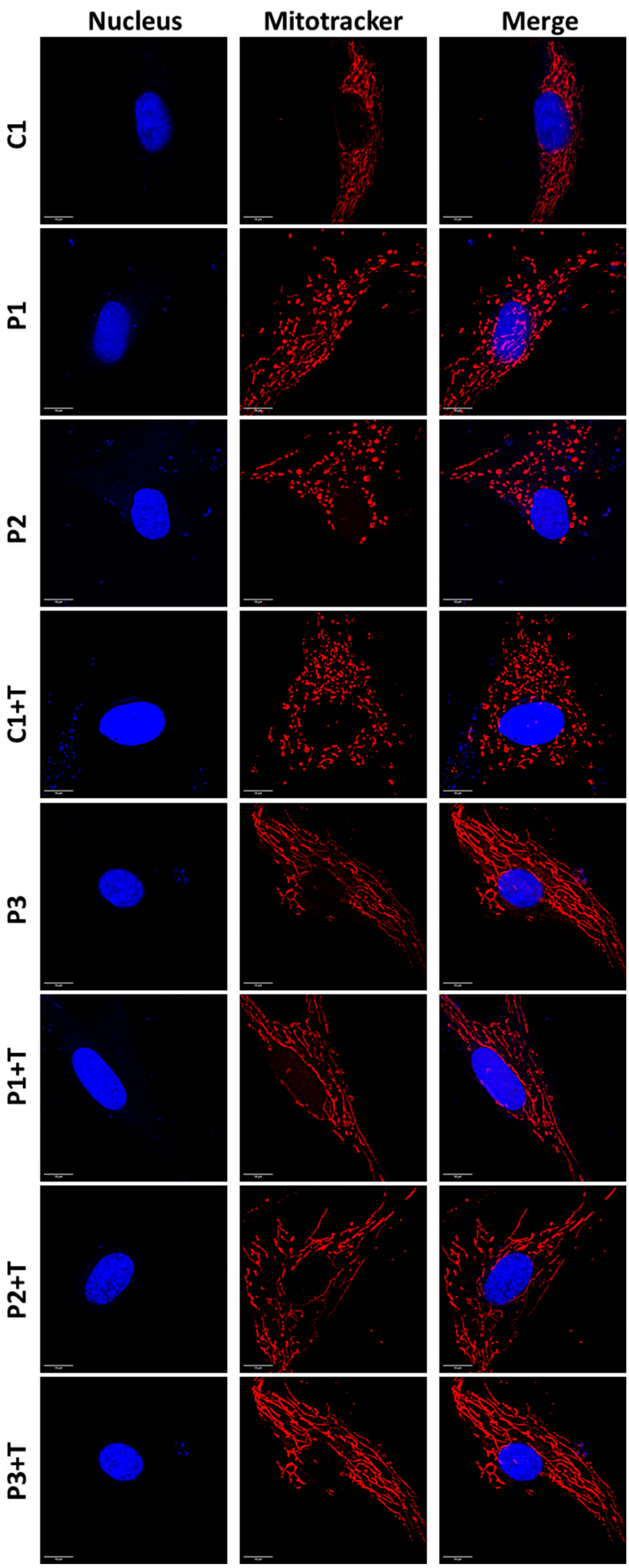

Figure S4. Images of Mitotracker^{TM} Deep Red FM. Control (C1) and mutant (P1, P2, P3) cells were treated with 10 µM of polydatin and nicotinamide for seven days (+T). Then, fibroblasts were stained with Mitotracker^{TM} Deep Red FM 100 nM for 45 minutes. Nuclei were visualized by DAPI staining. Images were acquired using a DeltaVision microscope. Scale bar = 20 µm.

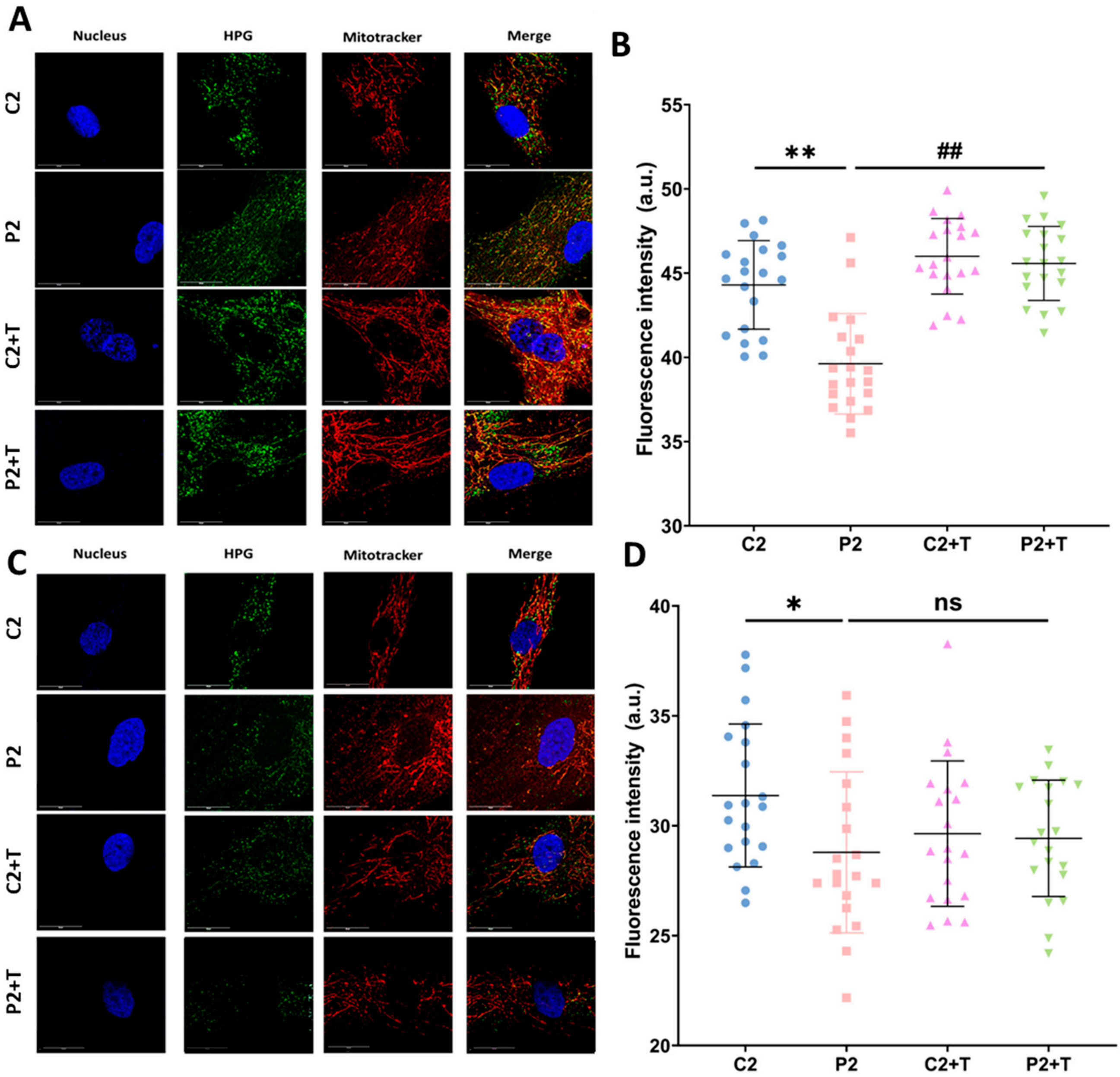

Figure S5. Cell protein synthesis. Control (C2) and mutant (P2) fibroblasts were subjected to polydatin and nicotinamide at 10 μM for seven days (+T). (**A**) Total protein synthesis. Cells were incubated with Mitotracker™ DeepRed FM for 45 minutes and then with HPG 488 Alexa Fluor for 30 minutes. (**B**) Quantification of fluorescence intensity of total protein synthesis. (**C**) Cytosolic protein synthesis. Fibroblasts were treated with chloramphenicol 150 μg/mL for 30 minutes to inhibit mitochondrial protein synthesis. After that, cells were incubated with Mitotracker™ DeepRed FM for 45 minutes and HPG 488 Alexa Fluor for 30 minutes. (**D**) Quantification of fluorescence intensity of cytosolic protein synthesis. Representative images were acquired using a Zeiss880 'Airyscan' microscope. At least 30 images were taken from each condition and experiment. *$p<0.05$, **$p<0.01$ between control and patient fibroblasts. $^{\#\#}p<0.01$ between untreated and treated *GFM1* fibroblasts. Scale bar = 20 μm. a.u.: arbitrary units. ns: not significant.

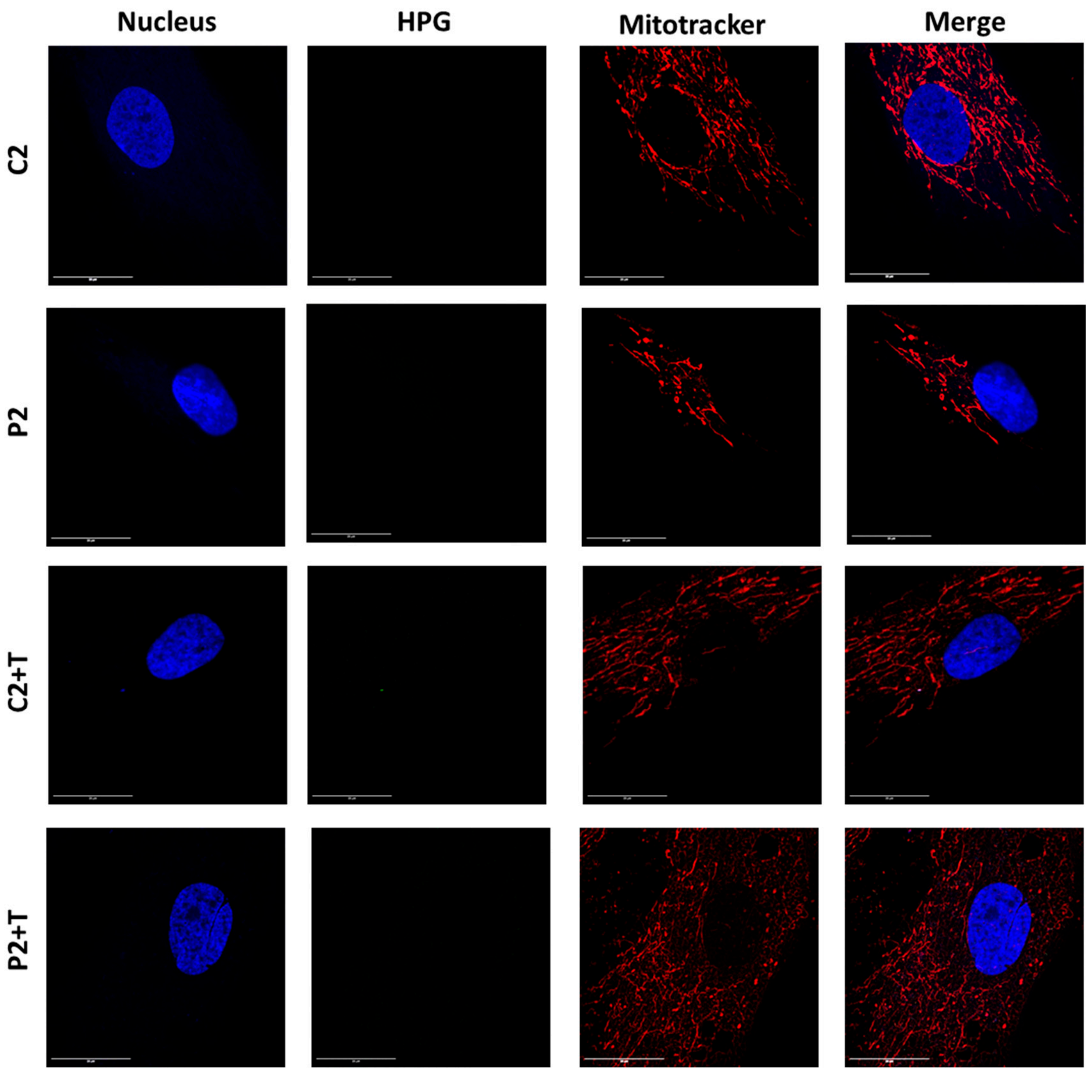

Figure S6. Negative control of cell protein synthesis. Control (C2) and mutant (P2) fibroblasts were treated with polydatin and nicotinamide at 10 µM for seven days (+T). After that, cells were treated with cycloheximide 50 µg/mL and chloramphenicol 150 µg/mL for 30 minutes. Then, fibroblasts were incubated with Mitotracker™ DeepRed FM for 45 minutes and HPG 488 Alexa Fluor for 30 minutes. Representative images were acquired using a Zeiss880 'Airyscan' microscope. Scale bar = 20 µm.

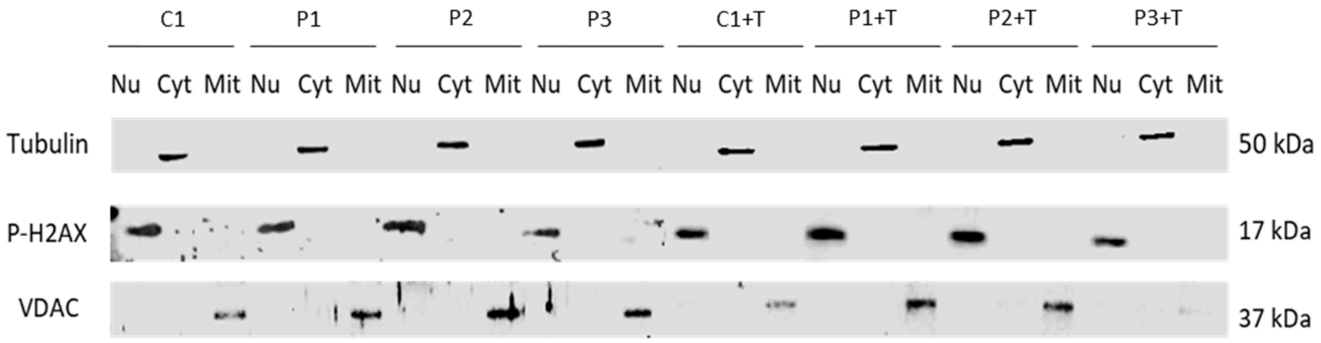

Figure S7. Purity of cellular fractions. Tubulin was used as cytoplasmic protein marker, P-H2AX was used as nuclear protein marker and VDAC as mitochondrial protein marker. C1: control cells. P1, P2, P3: mutant cells. T: treatment with polydatin and nicotinamide at 10 µM for seven days.

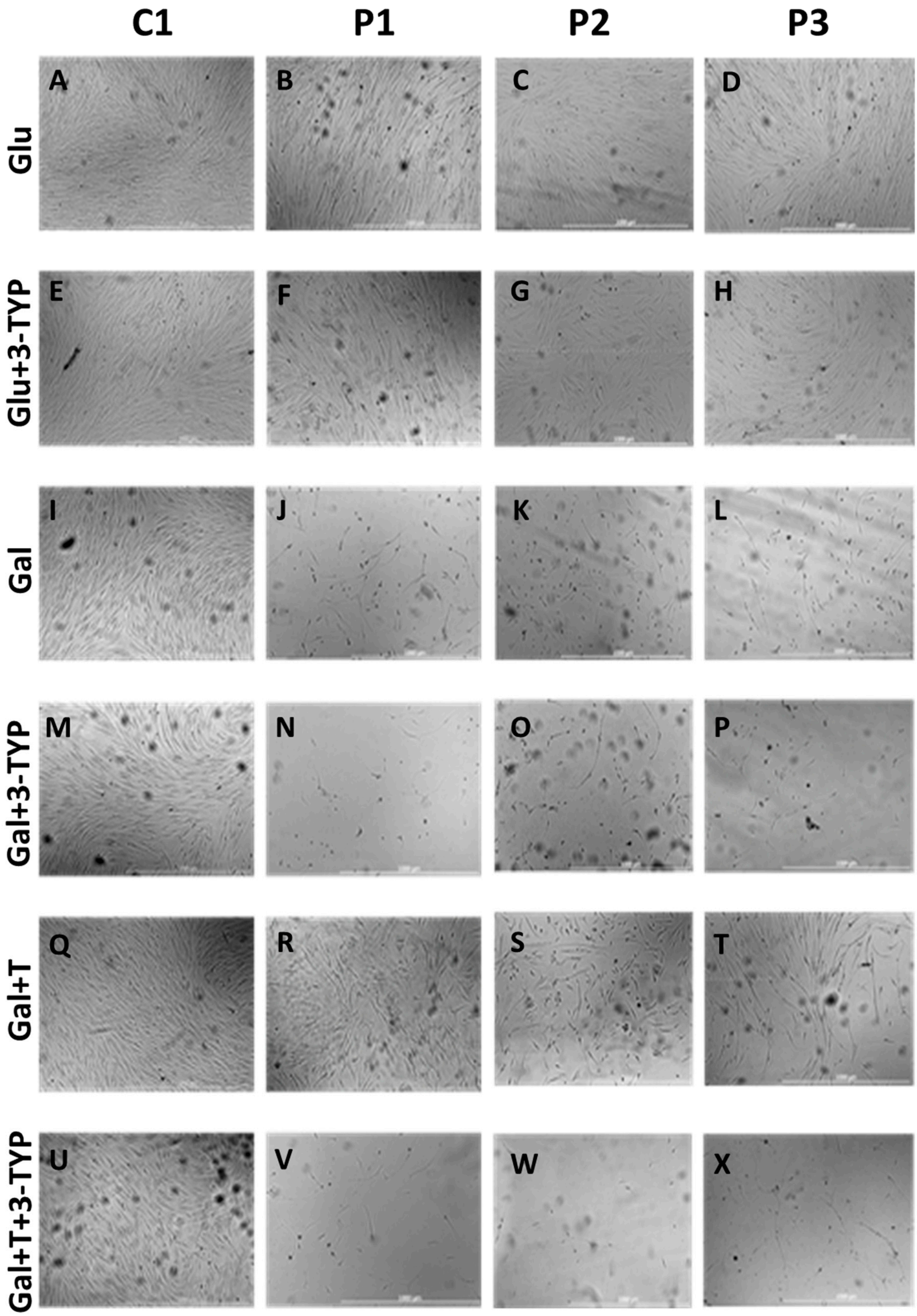

Figure S8. Effect of SIRT3 inhibition by 3-TYP on galactose screening. Control (C1) and mutant (P1, P2, P3) cells were initially seeded in glucose-rich medium and treated either with polydatin and nicotinamide at 10 µM (T) or T along with 32 nM 3-TYP for 3 days. After that, the glucose medium was replaced with galactose medium, and both treatment and 3-TYP were refreshed. Control fibroblasts grew normally in all experimental conditions **(A, E, I, M, Q, U)**, but patients' cells experienced cell death in galactose medium **(J, K, L)**, although they were able to survive in galactose medium with the treatment **(R, S, T)**. In the presence of 3-TYP, the three patient lines died in galactose medium even when they were treated **(V, W, X)**. Scale bar = 1000 µm.

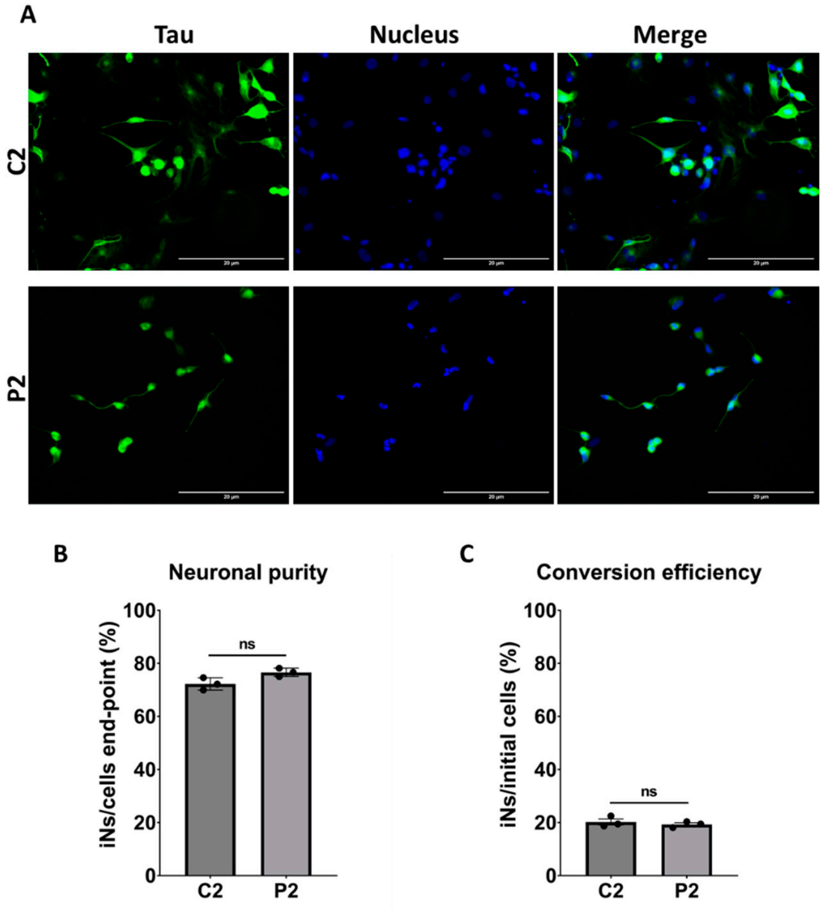

Figure S9. iNs generated by direct reprogramming. (**A**) Representative images of control (C2) and patient (P2) iNs stained against Tau. Undifferentiated cells showed only DAPI staining for the nucleus. (**B**) Neuronal purity, number of Tau+ cells over the total cells. (**C**) Conversion efficiency, number of Tau+ cells over the total of cells seeded at the beginning of the assay. Scale bar = 20 µm. ns: not significant.
